# Household food insecurity and symptoms of neurologic disorder in Ethiopia: An observational analysis

**DOI:** 10.1186/1471-2458-10-802

**Published:** 2010-12-31

**Authors:** Abdulrahman M El-Sayed, Craig Hadley, Fasil Tessema, Ayelew Tegegn, John A Cowan, Sandro Galea

**Affiliations:** 1 Department of Epidemiology, Columbia University, New York, NY, USA; 2 Columbia University College of Physicians and Surgeons, New York, NY, USA; 3 Department of Public Health, University of Oxford, Oxford, UK; 4 Department of Anthropology, Emory University, Atlanta, GA, USA; 5 Jimma University, Jimma, Ethiopia; 6 Department of Neurosurgery, University of Michigan Medical School, Ann Arbor, MI, USA

## Abstract

**Background:**

Food insecurity (FI) has been shown to be associated with poor health both in developing and developed countries. Little is known about the relation between FI and neurological disorder. We assessed the relation between FI and risk for neurologic symptoms in southwest Ethiopia.

**Methods:**

Data about food security, gender, age, household assets, and self-reported neurologic symptoms were collected from a representative, community-based sample of adults (N = 900) in Jimma Zone, Ethiopia. We calculated univariate statistics and used bivariate chi-square tests and multivariate logistic regression models to assess the relation between FI and risk of neurologic symptoms including seizures, extremity weakness, extremity numbness, tremors/ataxia, aphasia, carpal tunnel syndrome, vision dysfunction, and spinal pain.

**Results:**

In separate multivariate models by outcome and gender, adjusting for age and household socioeconomic status, severe FI was associated with higher odds of seizures, movement abnormalities, carpal tunnel, vision dysfunction, spinal pain, and comorbid disorders among women. Severe FI was associated with higher odds of seizures, extremity numbness, movement abnormalities, difficulty speaking, carpal tunnel, vision dysfunction, and comorbid disorders among men.

**Conclusion:**

We found that FI was associated with symptoms of neurologic disorder. Given the cross-sectional nature of our study, the directionality of these associations is unclear. Future research should assess causal mechanisms relating FI to neurologic symptoms in sub-Saharan Africa.

## Background

Household food insecurity (FI) has been defined as limited or uncertain availability of nutritionally adequate and safe foods or limited or uncertain ability to acquire acceptable foods in socially acceptable ways [1,2]. In wealthy countries, it has been estimated that 11.1% of households are food insecure, with FI higher than 35% among the poor [1]. FI and related indicators, food insufficiency and hunger, have been associated with decreased adult caloric intake [3-5], psychosocial dysfunction in children [6], overweight in young children [7], increased body weight in women [8,9], sociofamilial dysfunction [10], and lower self-reported physical and mental health [11-14].

FI, food insufficiency, and hunger are generally thought to be substantially more prevalent in poorer countries than they are in richer countries. Although the literature about the consequences of FI is sparse in less wealthy countries, the extant evidence suggests that the consequences of FI extend well beyond undernutrition [15,16]. Ethnographic and epidemiologic studies from sub-Saharan Africa (SSA) suggest that FI is associated with poor mental health [15,16]. Hadley and Patil showed that household FI was associated with symptoms of anxiety and depression in rural Tanzania [15], and that seasonal increases in FI were associated with increases in symptoms of depression and anxiety [16]. Hadley and colleagues showed that FI was associated with higher symptoms of depression, anxiety, and post-traumatic stress [17].

One of the potential correlates of FI is neurologic dysfunction (ND). It is estimated that 1 in 9 people worldwide die of a ND, and that ND accounts for 28% of years lived with disability [18]. Several studies have considered the prevalence of ND in Ethiopia. Tamrat and colleagues [19] found that the prevalence of all-cause disability in three rural areas in northern Ethiopia was 4.9%. In particular, the prevalence of epileptic seizure was 0.7%, of vision dysfunction was 1.5% and of walking difficulty was 1.7%. In a similar study, Fitaw and Boersma [20] assessed the prevalence of disability in one urban and three rural areas in northwestern Ethiopia. They found that the prevalence of disability was 3.8%; 47.0% of those had lower locomotive disability, 28.6% had vision dysfunction, 16.1% had upper motor disability, 10.3% had cognitive impairments, and 8.3% had hearing loss, respectively. Tekle-Haimanot and colleagues [21] assessed the incidence of specific neurologic diagnoses in a rural population in central Ethiopia. They found high incidence of the following disorders: epilepsy (520/100,000), postpoliomyelitis paralysis (240/100,000), mental retardation (170/100,000), peripheral neuropathy (150/100,000) and deaf-mutism (130/100,000). The incidence of hemiparesis was 62/100,000, that of cerebral palsy was 20/100,000, 16/100,000 for optic atrophy, 12/100,000 for perceptive deafness, 10/100,000 for tropical spastic paraparesis, 7/100,000 for Parkinson's disease and 5/100,000 each for motor neuron disease, ataxia, and chorea/athetosis.

Although there is a paucity of research about specific social and demographic determinants of ND, both behavioral, as well as physiological studies suggest that malnutrition in early neurologic development is a risk factor for ND in later life [22,23]. Considering the determinants of ND in less wealthy countries, the World Health Organization (WHO) suggested that malnutrition, perinatal complications, and advanced parasitic infection are among the most important worldwide determinants of ND. Because structural causes underlying some of the leading risk factors for disabling disorders of the nervous system, such as malnutrition, poor sanitation, and lack of healthcare access are most prevalent in less wealthy countries, populations in these countries are especially at risk [18,24].

In light of the extant research, it is plausible that FI could be a determinant of ND. Alternatively, it is also plausible that those with ND are more likely to be disabled and therefore, less capable of providing themselves and their families with a consistent food supply. This is especially true in rural SSA where subsistence agriculture is the primary means of food accrual. In this analysis, we use data from the Gilgel Gibe Growth and Development Study (GGGDS), a population-representative sample in southwest Ethiopia to assess the relation between FI and symptoms of ND, including spinal pain, extremity weakness, extremity numbness, seizure, vision dysfunction, aphasia, tremors/ataxia, carpal tunnel syndrome, and spinal pain.

## Methods

### Sample

This study took place in Jimma zone, southwest Ethiopia in the Gilgel Gibe area outside of Jimma Town. This is a predominantly rural area where the primary occupation is subsistence agriculture and FI is chronic.

The Gilgel Gibe Growth and Development Study (GGGDS) is a cohort study of families in the Gilgel Gibe Field Research Center that is concerned with adult mental health, neurologic health, and child development. The study involves questionnaire and anthropometric information collected from the parents, and developmental assessments conducted on their children. We report here on baseline information collected from the parents.

The baseline cohort for the GGGDS was a random sample of households that had a child between the ages of 3-24 months from the universe of all births in Gilgel Gibe in the two years prior to the estimated start date of the survey (~2000 births). We sampled 550 households at baseline. From these 79 households were not included because the children could not be located, had died, or had moved from the study area. An additional 20 households were excluded because the father was not living in the household or could not be located. Thus, the overall response rate was 82%.

A structured questionnaire was developed and administered to participants by 9 trained interviewers. Questionnaires and consent documents were developed in English then translated and back translated by native speakers into the two dominant languages in the study area: Amharic and Affan Oromo. Households were visited and all of the participants were interviewed in their house in a private area. Husbands and wives were separately interviewed using questionnaires developed specifically for men and women (refer to additional files 1 and 2 for survey questionnaires used among women in English and Amharic, respectively, and additional files 3 and 4 for questionnaires used among men in English and Amharic, respectively). Written informed consent was obtained from all participants. The Institutional Review Boards of the University of Michigan and Jimma University reviewed and approved the study protocol.

### Survey Domains

Household-level FI was measured using a seven-item scale based on those used and validated previously in diverse settings in developing countries [25-27]. Women and men were separately asked whether because food ran out or they did not have enough money to buy food in the last three months, they: (1) worried about running out of food, (2) ran out of food, (3) reduced variety of food for children, (4) children did not have enough to eat, (5) if the respondent or other adult did not eat enough, (6) the respondent spent the whole day without food, and/or (7) the household ever had to ask others for food or money to buy food. We were most interested in household food security. We therefore analyzed internal consistency (Cronbach's Alpha 0.93), and responses of the husband and wife were summed. Maternal and paternal summed scores were also highly correlated (p < 0.01) so the average was taken to represent the household's food insecurity situation. This score was divided into three food insecurity categories for the purposes of analysis: secure (score of 0-2), moderate food insecurity (score of 3-5), and severe food insecurity (score of 6-7).

Household socioeconomic status (SES) was measured through an asset index, as is standard in low-income countries [28]. The asset index was also used to measure SES in the Ethiopia Demographic and Health Survey in 2005 [29]. A set of questions about possession of material assets was asked of each household, including whether the household possessed household electricity, a television, a radio, a phone/mobile, and/or a tapedeck/VHS/DVD player. The number of items in household possession was summed and household socioeconomic status was categorized based on whether the household was above or below the median asset ownership, which was one of the above assets per household in this sample. Women provided corresponding responses to some of the material items and these were highly associated with their partners' responses. We therefore used men's responses as estimates of the household SES.

Additionally, we controlled for the effects of age (in years; age was subdivided into three categorical variables). Information on educational status was not collected since it is known to be universally very low in the study population [17].

### Dependent Variables

The neurologic disorders screening tool was derived from the World Health Organization research protocol for measuring the prevalence of neurologic disorders in developing countries [30]. A variation of this instrument was used successfully in a suburb of Addis Abba, the capital city of Ethiopia [31]. The survey instrument included questions about neurologic symptoms including: back pain, neck pain, hand/foot numbness, arm/leg weakness, seizures, diplopia, temporary blindness, difficulty speaking, movement abnormalities while still, movement abnormalities while intending movement, and carpal tunnel syndrome. Respondents were asked if they had experienced any symptom in the past week. Answers were coded dichotomously with a 1 indicating a "yes" answer, and a zero indicating a "no" answer. Similar symptoms were aggregated for the purpose of this analysis. The neurologic indicators of interest were: seizures (respondents reporting having had a seizure in the past week), extremity weakness (respondents reporting having had either arm/hand or leg/foot weakness in the past week), extremity numbness (respondents reporting having had either arm/hand numbness or leg/foot numbness in the past week), movement abnormalities (respondents reporting movement abnormalities either while still or while intending movement), difficulty speaking (respondents reporting having experienced either temporary or permanent difficulty speaking in the past week), carpal tunnel (respondents reporting hand pain that awoke them from sleep in the past week), vision dysfunction (respondents reporting having had diplopia or temporary vision loss in the past week), and spinal pain (respondents reporting having had back or neck pain in the past week). We aggregated our indicators to create an indicator of ND comorbidity (respondents reporting two or more NDs in the past week).

### Statistical Methods

Univariate statistics were used to describe the sample and bivariate chi-square tests were used to assess relations between each of the independent variables and outcomes of interest separately by gender. We used bivariate logistic regression to assess unadjusted associations between FI and neurologic symptoms by gender, and used multivariate logistic regression to adjust for age, and household assets. The criterion for statistical significance was set at α = 0.05. All statistical tests were carried out using SAS 9.2.

## Results

Complete data were available for 900 individuals (i.e. 450 men, and 450 women) for analysis. The majority of the sample (61.8%) was between the ages of 20 and 30. According to the median cut-off of the asset index, 47.3% of the sample was classified as having assets below the median number reported. Severe food insecurity was prevalent among 24.7% of the sample.

Overall, vision dysfunction was the most prevalent aggregate of symptoms with 60.9% of the sample reporting diplopia or temporary vision loss in the last week. Seizure was the least prevalent ND with only 6.3% of the sample reporting a seizure in the past week. 48.1% of the sample reported symptoms falling within two or more symptom clusters within the past week.

Table 1 shows univariate statistics and bivariate chi-square tests between FI and other covariates and neurological disorders among women in Jimma Zone. In chi-square models, FI was significantly associated with higher prevalence of each outcome of interest (p < 0.050) except extremity weakness (p = 0.282), difficulty speaking (p = 0.092), and spinal pain (0.123). Table 2 shows the same information among men in Jimma Zone. In chi-square models, FI was significantly associated with higher prevalence of all outcomes of interest (p < 0.050) except extremity weakness (p = 0.256).

**Table 1:** Univariate and bivariate statistics of all neurologic symptom aggregates and covariates among 450 adult women in Jimma zone, Ethiopia

	Total	Seizures		Extremity Weakness		Extremity Numbness		Movement Abnormalities		Difficulty Speaking		Carpal Tunnel		Vision Dysfunction		Spinal Pain		Comorbid Disorders	
	%	%	p	%	p	%	p	%	p	%	p	%	p	%	p	%	p	%	p
Overall	100	5.3	--	29.1	--	23.8	--	18.0	--	5.3	--	12.9	--	42.7	--	11.6	--	42.0	--
																			
Food Insecurity			0.001		0.282		0.038		< 0.001		0.092		< 0.001		< 0..001		0.123		< 0.001
Secure	53.8	3.3		28.9		20.3		9.9		4.1		8.7		36.4		9.9		36.0	
Moderate	22.9	2.9		24.3		33.0		23.3		3.9		9.7		40.8		9.7		38.8	
Severe	23.3	12.4		34.3		22.9		31.4		9.5		25.7		59.1		17.1		59.1	
Age			0.502		0.053		0.005		0.786		0.351		< 0.001		0.004		0.091		0.007
< 30	8.4	9.7		19.4		25.8		16.1		0.0		3.2		22.6		9.7		32.3	
30-40	71.6	5.3		30.6		20.8		17.0		6.04		11.3		41.9		9.8		40.8	
> 40	20.0	4.1		26.3		39.2		20.3		6.8		27.0		56.8		18.9		59.5	
Asset Index*			0.491		0.677		0.419		0.164		0.267		0.118		0.551		0.059		0.627
high	52.7	4.6		30.0		25.3		15.6		4.2		10.6		41.4		8.89		40.9	
low	47.3	6.1		28.2		22.1		20.7		6.6		15.5		44.1		14.6		43.2	

* A set of material assets was asked of each household, including the possession of household electricity, a television, a radio, a phone/mobile, and/or tapedeck/VHS/DVD player; items were summed and household's socioeconomic status was categorized based on whether the household was above or below the median asset ownership, which was one of the above assets per household.

**Table 2:** Univariate and bivariate statistics of all neurologic symptom aggregates and covariates among 450 adult men in Jimma zone, Ethiopia

	Total	Seizures		Extremity Weakness		Extremity Numbness		Movement Abnormalities		Difficulty Speaking		Carpal Tunnel		Vision Dysfunction		Spinal Pain		Comorbid Disorders	
	%	%	p	%	p	%	p	%	p	%	p	%	p	%	p	%	p	%	p
Overall	100	5.1	--	28.0	--	22.9	--	17.2	--	5.1	--	12.5	--	40.9	--	11.2	--	40.3	--
																			
Food Insecurity			0.003		0.256		0.009		< 0.001		0.015		< 0.001		< 0.001		0.003		< 0.001
Secure	53.5	2.5		28.1		18.2		9.9		3.3		7.4		34.7		9.9		32.6	
Moderate	18.1	4.9		24.4		31.7		19.5		3.7		7.3		35.2		4.9		39.0	
Severe	28.3	10.9		34.4		29.7		32		10.2		27.3		63.3		19.5		61.7	
Age			0.183		< 0.001		< 0.001		0.019		0.367		< 0.001		< 0.001		0.134		< 0001
< 20	39.4	2.6		15.8		15.1		11.2		4.0		4.6		23.0		8.6		23.7	
20-30	44.8	4.1		34.1		27.8		21.4		6.9		15.0		53.2		13.3		50.3	
31-40	15.8	8.2		41.0		39.4		24.6		3.3		23.0		42.6		18.0		54.1	
> 40																			
Asset Index*			0.373		0.812		0.832		0.074		0.19		0.043		0.159		0.012		0.196
high	54.7	4.3		27.5		23.3		14.3		3.9		9.7		38.0		8.1		37.6	
low	45.3	6.1		28.5		22.4		20.6		6.5		15.9		44.4		15.0		43.5	

* A set of material assets was asked of each household, including the possession of household electricity, a television, a radio, a phone/mobile, and/or tapedeck/VHS/DVD player; items were summed and household's socioeconomic status was categorized based on whether the household was above or below the median asset ownership, which was one of the above assets per household.

Tables 3 shows bivariate and multivariate regression models of each outcome of interest by FI and other covariates among men and women in our sample. Among women, severe FI was associated with higher odds of seizures, movement abnormalities, carpal tunnel, vision dysfunction, and spinal pain in both unadjusted and adjusted models. Severe FI was also associated with higher odds of having comorbid disorders in both unadjusted and adjusted models. Moderate FI was only associated with extremity numbness and movement abnormalities in both unadjusted and adjusted models.

**Table 3:** Odds ratios and 95% confidence intervals from unadjusted and adjusted multivariable logistic regression           models of neurologic symptom aggregates by food insecurity among 450 women in Jimma zone, Ethiopia

	Seizures		Extremity Weakness		Extremity Numbness		Movement Abnormalities		Difficulty Speaking		Carpal Tunnel		Vision Dysfunction		Spinal Pain		Comorbid Disorders	
Food Insecurity																		
Secure	Ref	Ref	Ref	Ref	Ref	Ref	Ref	Ref	Ref	Ref	Ref	Ref	Ref	Ref	Ref	Ref	Ref	Ref
Moderate	1.13 (0.51-2.50)	0.98 (0.44-2.21)	0.79 (0.46-1.34)	0.76 (0.44-1.31)	1.94 (1.16-3.25)	2.00 (1.17-3.41)	2.76 (1.48-5.14)	2.66 (1.42-5.00)	0.94 (0.29-3.06)	0.86 (0.26-2.83)	1.07 (0.42-2.79)	1.01 (0.38-2.66)	1.1 (0.65-1.86)	1.08 (0.63-1.86)	0.51 (0.17-1.51)	0.47 (0.16-1.41)	1.48 (0.89-2.48)	1.46 (0.86-2.47)
Severe	3.64 (1.95-6.81)	3.31(1.74-6.39)	1.28 (0.79-2.09)	1.23 (0.74-2.03)	1.17 (0.67-2.03)	1.13 (0.64-1.99)	4.16 (2.31-7.51)	4.07 (2.25-7.38)	2.44 (0.99-6.06)	2.28 (0.91-5.73)	5.10 (2.75-9.42)	4.23 (2.24-7.98)	3.65 (2.34-5.69)	3.16 (1.99-5.01)	2.41 (1.31-4.41)	2.09 (1.12-3.92)	3.73 (2.40-5.82)	3.27 (2.07-5.18)
Age																		
< 30		Ref		Ref		Ref		Ref		Ref		Ref		Ref		Ref		Ref
30-40		1.70 (0.74-3.89)		2.13 (1.22-3.73)		0.99 (0.57-1.71)		0.88 (0.48-1.58)		2.37 (0.67-8.37)		0.36 (0.15-0.85)		0.33 (0.21-0.52)		0.90 (0.44-1.82)		0.41 (0.26-0.65)
> 40		4.67 (1.89-11.56)		3.48 (1.76-6.87)		2.41 (1.24-4.67)		0.95 (0.44-2.05)		2.46 (0.56-10.73)		1.75 (0.85-3.62)		0.69 (0.38-1.23)		1.69 (0.78-3.69)		1.34 (0.75-2.40)
Asset Index*																		
high		Ref		Ref		Ref		Ref		Ref		Ref		Ref		Ref		Ref
low		1.51 (0.84-2.71)		0.95 (0.62-1.44)		0.75 (0.48-1.18)		1.19 (0.72-1.97)		1.59 (0.68-3.71)		1.35 (0.75-2.42)		1.1 (0.74-1.64)		1.73 (0.95-3.15)		1.03 (0.69-1.54)

* A set of material assets was asked of each household, including the possession of household electricity, a television, a radio, a phone/mobile, and/or tapedeck/VHS/DVD player; items were summed and household's socioeconomic status was categorized based on whether the household was above or below the median asset ownership, which was one of the above assets per household.

Table 4 shows bivariate and multivariate regression models of each outcome of interest by FI and other covariates among men in our sample. Among men, severe FI was associated with higher odds of seizures, extremity numbness, movement abnormalities, difficulty speaking, carpal tunnel, and vision dysfunction in both unadjusted and adjusted models. Severe FI was also associated with higher odds of having comorbid disorders in both unadjusted and adjusted models. Moderate FI was only associated with extremity numbness and movement abnormalities in both unadjusted and adjusted models.

**Table 4:** Odds ratios and 95% confidence intervals from unadjusted and adjusted multivariable logistic regression            models of neurologic symptom aggregates by food insecurity among 450 men in Jimma zone, Ethiopia

	Seizures		Extremity Weakness		Extremity Numbness		Movement Abnormalities		Difficulty Speaking		Carpal Tunnel		Vision Dysfunction		Spinal Pain		Comorbid Disorders	
Food insecurity																		
Secure	Ref	Ref	Ref	Ref	Ref	Ref	Ref	Ref	Ref	Ref	Ref	Ref	Ref	Ref	Ref	Ref	Ref	Ref
Moderate	2.19 (0.60-7.95)	2.12 (0.58-7.73)	0.92 (0.52-1.64)	0.89 (0.49-1.60)	2.30 (1.31-4.06)	2.30 (1.29-4.11)	2.40 (1.21-4.79)	2.33 (1.17-4.66)	1.21 (0.31-4.65)	1.17 (0.30-4.54)	1.13 (0.51-2.50)	0.98 (0.44-2.21)	1.21 (0.75-1.93)	1.16 (0.72-1.88)	1.94 (1.16-3.25)	0.84 (0.38-1.86)	1.13 (0.70-1.82)	1.08 (0.66-1.76)
Severe	5.24 (1.96-13.98)	4.64 (1.70-12.67)	1.49 (0.95-2.36)	1.29 (0.80-2.07)	2.09 (1.27-3.45)	1.97 (1.17-3.31)	4.67 (2.67-8.19)	4.18 (2.36-7.43)	3.59 (1.45-8.90)	3.29 (1.29-8.38)	3.64 (1.95-6.81)	3.31 (1.74-6.29)	2.52 (1.58-4.03)	2.42 (1.51-3.90)	1.17 (0.67-2.03)	1.67 (0.85-3.27)	2.57 (1.61-4.11)	2.46 (1.53-3.97)
Age																		
< 30		Ref		Ref		Ref		Ref		Ref		Ref		Ref		Ref		Ref
30-40		0.57 (0.18-1.78)		0.42 (0.25-0.69)		0.62 (0.36-1.06)		0.67 (0.36-1.24)		0.79 (0.30-2.13)		1.70 (0.74-3.89)		1.34 (0.84-2.14)		0.92 (0.44-1.90)		1.39 (0.86-2.22)
> 40		1.42 (0.48-5.15)		1.49 (0.84-2.65)		2.13 (1.17-3.88)		1.36 (0.68-2.70)		0.48 (0.11-2.18)		4.67 (1.89-11.56)		2.31 (1.25-4.27)		1.88 (0.81-4.39)		2.83 (1.52-5.26)
Asset Index*																		
high		Ref		Ref		Ref		Ref		Ref		Ref		Ref		Ref		Ref
low		1.11 (0.47-2.60)		0.95 (0.63-1.45)		0.80 (0.51-1.26)		1.23 (0.74-2.04)		1.49 (0.63-3.50)		1.51 (0.84-2.71)		1.07 (0.72-1.57)		1.73 (0.95-3.16)		1.05 (0.71-1.56)

* A set of material assets was asked of each household, including the possession of household electricity, a television, a radio, a phone/mobile, and/or tapedeck/VHS/DVD player; items were summed and household's socioeconomic status was categorized based on whether the household was above or below the median asset ownership, which was one of the above assets per household.

Table 5 shows comorbidity between outcomes of interest among the full sample. Generally, there was high comorbidity between outcomes. In particular, all outcomes were highly comorbid with vision dysfunction. Alternatively, outcomes were not often comorbid with seizures.

**Table 5:** Comorbidity (in percent) between neurological disorders among 900 women and men in Jimma zone, Ethiopia*

	Seizures	Extremity weakness	Extremity numbness	Vision dysfunction	Difficulty speaking	Movement abnormalities	Spinal pain	Carpal Tunnel
Seizures	--	45.8	41.7	79.2	12.5	37.5	37.5	37.5
Extremity weakness	8.3	--	38.6	62.9	9.1	30.3	18.9	17.4
Extremity numbness	9.3	47.2	--	52.8	7.4	39.8	16.7	27.8
Vision dysfunction	9.8	43.0	29.5	--	9.8	34.7	18.1	20.7
Difficulty speaking	12.5	50.0	33.3	79.2	--	54.2	37.5	45.8
Movement abnormalities	11.1	49.4	53.1	82.7	16.1	--	24.7	39.5
Spinal pain	17.0	47.2	34.0	66.0	17.0	37.7	--	35.9
Carpal Tunnel	15.3	40.0	50.9	67.8	18.6	54.2	32.2	--

*Numbers represent the percentage of those in each row with each comorbidity, by column

## Discussion

Using data from a population-based representative sample of households in a rural area in Ethiopia, we found that FI was associated with a range of neurologic symptoms including self-reported seizure, extremity numbness, movement abnormalities, speaking difficulties, carpal tunnel syndrome, spinal pain, and vision dysfunction. FI was also associated with a higher prevalence of ND symptom comorbidity. We found that FI was generally more predictive of ND symptoms among men than among women. Finally, ND symptoms in this population were highly comorbid.

We know of no other studies that have assessed the relation between FI and risk for neurologic symptoms. However, our findings are consistent with literature documenting associations between FI and adverse health outcomes in both wealthy and less wealthy countries. For example, in a cross-sectional analysis of 724 randomly chosen low-income single women participating in welfare in the state of Michigan, USA, Siefert and colleagues [11] found that food insufficiency was associated with poor or fair self-rated health and DSM-III criteria major depressive symptoms. In a sample of 1,006 men and women in the rural US, Pheley and colleagues [12] found that FI was associated with poorer health measures before and after adjustment for demographic and healthcare access variables. Using data from the National Population Health Survey in Canada, food insufficiency was associated with self-reported heart disease, diabetes, hypertension, food allergies, and overweight among men, as well as poorer mental and social health [13]. Among 1488 households in a rural region of the United States, FI was associated with lower self-reported physical and mental health as well as poorer health measures [32]. Another study, by Gao and colleagues, found that FI was associated with lower cognitive function in Hispanics living in the US [33]. Other studies, summarized earlier, have also shown there to be associations between FI and health in SSA [15,17].

One of the central challenges in the literature on this topic is the directionality of the association between FI and adverse health. Helping clarify this question, three longitudinal cohort studies provide evidence that is suggestive that FI or food insufficiency is a causal determinant of worse mental and physical health. Siefert and colleagues [14] found in a longitudinal analysis of 753 female public assistance recipients in the US that persistent, recurrent food insufficiency over two years was associated with higher limitations in physical functioning as compared to having only experienced FI in the present period. Heflin and colleagues [34] found in the same cohort that increases in food insufficiency predicted increases in symptoms of major depression over a three year study period. In Tanzania, Hadley and Patil [16] found that among 173 caretakers, seasonal variation in FI predicted changes in symptoms of depression and anxiety. Biological evidence also suggests that malnutrition during early neural development can cause ND in later life [18,24]. Conversely, however, it is also plausible that persons suffering from NDs are more likely to be disabled, and therefore unable to provide themselves with a consistent food supply. This is especially likely to be true in economies that are based heavily on subsistence agriculture. Further research will need to disentangle the causal relationship between FI and ND.

Our work also highlights the current paucity of and need for quality instruments for assessing the burden of ND in the rural SSA setting. We found considerable discordance between the prevalences of common symptoms of ND in our sample and those reported in comparable studies. For example, we found that 60.9% (not shown) of respondents reported vision dysfunction, which compares to 1.5% and 28.6% in comparable studies [19,35]. Moreover, we found that 6.3% (not shown) of our sample reported seizure compared 0.7% in a comparable study [36]. Although there is no gold standard in the field, given that the prevalences of common symptoms of ND in our sample appear higher than those reported in similar studies, it is likely that our instrument overestimated the prevalences of common ND in our study population.

There are several limitations that should be considered when interpreting our findings. First, we recorded symptoms that could potentially have neurologic causes, rather than specific diagnoses of ND, and it is impossible to differentiate between neurologic, orthopedic, or other causes of many of the symptoms included in our instrument. Moreover, symptoms were not confirmed by neurologists. Therefore, the prevalences reported here should be interpreted very cautiously; it is impossible to draw conclusions about the prevalence of specific diagnoses within this population or about the specific etiologies of individual symptoms. Of particular note, for example, 60.9% of our sample reported vision dysfunction. However, because there is a high prevalence of organic eye diseases that affect vision in Ethiopia [37,38], it is possible that a sizable proportion of the vision dysfunction in our population is not of neurologic etiology. Second, because we sampled only households with children, it is possible that our findings do not generalize to households without children. Third, our covariate set was limited and therefore, there may be residual confounding of the association between FI and each symptom aggregate. Of particular note, we did not collect data about dietary intake, and therefore, it is plausible that the observed associations could be confounded by malnutrition.

It has been estimated that 33% of people living in SSA are undernourished, a widely used indicator of FI. Unfortunately, in SSA, FI is likely to increase in the coming decades [39,40]. Moreover, the global burden of ND is estimated to rise significantly by 2020 [18]. Therefore, studies that explore the correlates of FI and ND in SSA are needed to inform clinicians and public health practitioners about how to better serve the needs of populations in this region. Investigators interested in the social causes and effects of ND could fruitfully explore different causal mechanisms that relate FI and ND. Other avenues for future research include assessing the relation between FI and specific ICD-10 diagnosable ND and exploring the associations between FI and ND in other geographic contexts. Finally, quality instruments for measuring ND in populations in SSA are deficient, and researchers interested in the epidemiology of ND in SSA might consider creating and validating such instruments for use in future studies.

## Conclusions

Among a population-based representative sample of households in a rural area in Ethiopia, FI was associated with several self-reported neurologic symptoms, including seizure, extremity numbness, movement abnormalities, speaking difficulties, carpal tunnel syndrome, spinal pain, and vision dysfunction, as well as higher prevalence of neurological symptom comorbidity. The directionality of these relations is unclear, and research about potential mechanisms relating FI and ND is needed. Finally, quality instruments for measuring ND in the SSA context are deficient, and research is needed to construct and validate such tools.

## Abbreviations

FI: Food insecurity; SSA: Sub-Saharan Africa; ND: Neurological Dysfunction; GGGDS: Gilgel Gibe Growth and Development Study; SES:Socioeconomic status.

## Competing interests

The authors declare that they have no competing interests.

## Authors' contributions

AME conceived the analysis, analyzed the data, and drafted the manuscript. CH conceived the study, coordinated data collection, and edited the manuscript. FT, AT, and JAC coordinated data collection and edited the manuscript. SG coordinated data collection, advised on data analysis, and edited the manuscript. Finally, all authors read and approved the final manuscript

## Pre-publication history

The pre-publication history for this paper can be accessed here:

http://www.biomedcentral.com/1471-2458/10/802/prepub

## Supplementary Material

Additional file 1 **Survey questionnaire for women and children in English**. The survey questionnaire used to obtain data about women in the analysis translated in English. Click here for file

Additional file 2 **Survey questionnaire for women and children in Amharic**. The survey questionnaire used to obtain data about women in the analysis in Amharic. Click here for file

Additional file 3 **Survey questionnaire for men in English**. The survey questionnaire used to obtain data about men in the analysis translated in English. Click here for file

Additional file 4 **Survey questionnaire for men in Amharic**. The survey questionnaire used to obtain data about men in the analysis in Amharic. Click here for file
