# Supplementary material for: Household food insecurity and symptoms of neurologic disorder in Ethiopia: An observational analysis
Source: BMC Public Health. 2010 Dec 31;10:802. doi: 10.1186/1471-2458-10-802 (PMC3027184; doi:10.1186/1471-2458-10-802)
Supplement: Additional file 1 — Survey questionnaire for women and children in English. The survey questionnaire used to obtain data about women in the analysis translated in English. [file 1471-2458-10-802-S1.DOC]

**Research project on “Child health, development and adult mental health”**

**Confidential: used only for research purposes**

| Name of interviewer | | Kebele name --------------- | | | Household ID ----------------- | | |
| --- | --- | --- | --- | --- | --- | --- | --- |
| Interviewer signature : | |
| Name of supervisors | | House number ----------- | | | Mother ID ---------------- | | |
| Supervisors signature : | |  | | | Child ID ------------------- | | |
| Outcome of first visit | 1.Completed 2. Not around home 3. Interrupted 4. Refused | |  | Date of appointment for other time visit | | | |
| Outcome of second visit | 1.Completed 2. Not around home 3. Interrupted 4. Refused | |  | Date of appointment for other time visit | | | |
| Outcome of third Visit | 1.Completed 2. Not around home 3. Interrupted 4. Refused | |  | Date of appointment for other time visit | | | |
| Date of interview date/month/ year | -------- /--------/----------- | | Time of start of interview | | | |  |
| Child sex | | | | | 0. Male | 1. Female |  |
| Is the child present: | | | | | 0. No | 1. Yes |  |

| **AGE APPROPRIATE DEVELOPMENTAL MILESTONES:** | |
| --- | --- |
| I am going to begin by asking you about your child. I will ask you whether or not your child is currently able to perform the following actions. I would like you to tell me whether or not your child is able to perform this action by him or herself; that is, without any help from you or others. | |
| 1. What is your child’s birthday? /months/ | -------- /--------/-----------  Date Month Year |
| 1. Is your child [months] old? | ------------------- months |

| **Personal social** | **Yes** | **No** |  | **Observed** | **Not observed** |  |
| --- | --- | --- | --- | --- | --- | --- |
| smile responsibly |  |  |  | 2 | 3 |  |
| smile spontaneously | 1 | 0 |  | 2 |  |  |
| work for toy |  |  |  | 2 | 3 |  |
| wave good bye | 1 | 0 |  | 2 |  |  |
| play ball with examiner |  |  |  | 2 | 3 |  |
| drinks from cup | 1 | 0 |  | 2 |  |  |
| imitates activities | 1 | 0 |  | 2 |  |  |
| help in the house | 1 | 0 |  | 2 |  |  |
| remove garment | 1 | 0 |  | 2 |  |  |
| put on clothing | 1 | 0 |  | 2 |  |  |
| wash hands | 1 | 0 |  | 2 |  |  |

| **Fine motor** | **Yes** | **No** |  | **Observed** | **Not observed** |  |
| --- | --- | --- | --- | --- | --- | --- |
| follow pas midline |  |  |  | 2 | 3 |  |
| follow 180 |  |  |  | 2 | 3 |  |
| grasp rattle |  |  |  | 2 | 3 |  |
| reaches object, toys |  |  |  | 2 | 3 |  |
| grasp small objects |  |  |  | 2 | 3 |  |
| bang two blocks |  |  |  | 2 | 3 |  |
| put block in cup |  |  |  | 2 | 3 |  |
| build a tower of 2 cubes |  |  |  | 2 | 3 |  |
| build a tower of 4 cubes |  |  |  | 2 | 3 |  |
| build a tower of 6 cubes |  |  |  | 2 | 3 |  |
| build a tower of 8 cubes |  |  |  | 2 | 3 |  |
| **Language** | **Yes** | **No** |  | **Observed** | **Not observed** |  |
| responds to bell |  |  |  | 2 | 3 |  |
| vocalizes | 1 | 0 |  | 2 |  |  |
| laughs | 1 | 0 |  | 2 |  |  |
| squeals | 1 | 0 |  | 2 |  |  |
| imitate speech sounds | 1 | 0 |  | 2 |  |  |
| say mama/dada non-specific | 1 | 0 |  | 2 |  |  |
| say mama/dad specific | 1 | 0 |  | 2 |  |  |
| say two words | 1 | 0 |  | 2 |  |  |
| say three words | 1 | 0 |  | 2 |  |  |
| say up to 6 words | 1 | 0 |  | 2 |  |  |
| name pictures |  |  |  | 2 | 3 |  |
| point body parts |  |  |  | 2 | 3 |  |
| point to four pictures |  |  |  | 2 | 3 |  |
| speech half understood |  |  |  | 2 | 3 |  |
| **Gross motor** | **Yes** | **No** |  | **Observed** | **Not observed** |  |
| left head |  |  |  | 2 | 3 |  |
| head up 45 degree |  |  |  | 2 | 3 |  |
| head up 90 degree |  |  |  | 2 | 3 |  |
| pull to sit |  |  |  | 2 | 3 |  |
| site without support |  |  |  | 2 | 3 |  |
| stand holding something |  |  |  | 2 | 3 |  |
| pull to stand |  |  |  | 2 | 3 |  |
| stand for 2 seconds |  |  |  | 2 | 3 |  |
| stand alone |  |  |  | 2 | 3 |  |
| walk well |  |  |  | 2 | 3 |  |
| walk backwards |  |  |  | 2 | 3 |  |
| run |  |  |  | 2 | 3 |  |
| kick ball forward |  |  |  | 2 | 3 |  |
| walk steps | 1 | 0 |  | 2 |  |  |
| throw ball overhead |  |  |  | 2 | 3 |  |
| jump by raising both legs together |  |  |  | 2 | 3 |  |

| **ARE GIVING PRACTICES**  Now I am going to ask you some questions about how your child eats. | | | | | | |
| --- | --- | --- | --- | --- | --- | --- |
|  | | No | | Yes |  | |
| 1. Did you ever breastfeed [NAME]? | | 0 → 5 | | 1 |  | |
| 1. How long after birth did you first put [NAME] to the breast? | Minutes |  | | | | |
|  | Hours |  | | | | |
|  | days |  | | | | |
|  | | No | Yes | | |  |
| 1. Before you breastfed for the very first time did you give [NAME] any liquid or food? | | 0 | 1 | | |  |
| 1. Since this time yesterday, have you breastfed [NAME]? | | 0 | 1 | | |  |

| HAS [NAME] **EVER** RECEIVED ANY OF THE FOLLOWING? | | | | | No | Yes |  |
| --- | --- | --- | --- | --- | --- | --- | --- |
| 1. Vitamins, mineral supplements, medicine | | | | | 0 | 1 |  |
| 1. Plain water | | | | | 0 | 1 |  |
| 1. Sweetened or flavored water | | | | | 0 | 1 |  |
| 1. Juice or teas | | | | | 0 | 1 |  |
| 1. Infant formula | | | | | 0 | 1 |  |
| 1. Tinned, powdered, or fresh milk (nonhuman) | | | | | 0 | 1 |  |
| 1. Any other liquids | | | | | 0 | 1 |  |
| 1. Any mushy, semi-solid, or solid foods | | | | | 0 | 1 |  |
| 1. ORS | | | | | 0 | 1 |  |
| 1. Any meat or fish? | | | | | 0 | 1 |  |
| 1. Has [NAME] had **DIARRHEA** in the last 2 weeks? | | | 0. No *(****If no, SKIP to Question 23***) | | | 1. Yes |  |
| **During** the last episode of diarrhea, did you offer [NAME] any of the following? | | | | | |  |  |
| 1. Breastmilk | | | | 0 = No | | 1 = Yes |  |
| 1. Other liquids | | | | 0 = No | | 1 = Yes |  |
| 1. Mushy (semisolid) or solid foods | | | | 0 = No | | 1 = Yes |  |
| 1. **During** [NAME]s diarrhea, did [NAME] breastfeed less than usual, the same, or more? | 1 = Less than usual | 2 = About the same | | 3 = More than usual | | 88 = NA |  |
| 1. **During** [NAME]’s diarrhea, did you offer him/her less food in **total**, about the same, or more food than usual? | 1 = Less than usual | 2 = About the same | | 3 = More than usual | | 88 = NA |  |
| 1. **During** [NAME]s diarrhea, did [NAME] Mushy (semisolid) or solid foods less than usual, the same, or more? | 1 = Less than usual | 2 = About the same | | 3 = More than usual | | 88 = NA |  |

| 1. Has [NAME] had **FEVER** in the last 2 weeks? | | | 0=No *(****If no, SKIP to Question 30***) | 1 = Yes |  |
| --- | --- | --- | --- | --- | --- |
| **During** the last episode of Fever , did you offer [NAME] any of the following? | | |  |  |  |
| 1. Breast milk | | | 0 = No | 1 = Yes |  |
| 1. Other liquids | | | 0 = No | 1 = Yes |  |
| 1. Mushy (semisolid) or solid foods | | | 0 = No | 1 = Yes |  |
| 1. **During** [NAME]s fever, did [NAME] breastfeed less than usual, the same, or more? | 1 = Less than usual | 2 = About the same | 3 = More than usual | 88 = NA |  |
| 1. **During** [NAME]’s fever, did you offer him/her less food in **total**, about the same, or more food than usual? | 1 = Less than usual | 2 = About the same | 3 = More than usual | 88 = NA |  |
| 1. **During** [NAME]s Fever, did [NAME] Mushy (semisolid) or solid foods less than usual, the same, or more? | 1 = Less than usual | 2 = About the same | 3 = More than usual | 88 = NA |  |

| 1. Has [NAME] had **Cough** in the last 2 weeks? | | | 0=No *(****If no, SKIP to Question 37***) | 1 = Yes |  |
| --- | --- | --- | --- | --- | --- |
| **During** the last episode of cough , did you offer [NAME] any of the following? | | |  |  |  |
| 1. Breast milk | | | 0 = No | 1 = Yes |  |
| 1. Other liquids | | | 0 = No | 1 = Yes |  |
| 1. Mushy (semisolid) or solid foods | | | 0 = No | 1 = Yes |  |
| 1. **During** [NAME]s cough did [NAME] breastfeed less than usual, the same, or more? | 1 = Less than usual | 2 = About the same | 3 = More than usual | 88 = NA |  |
| 1. **During** [NAME]’s cough, did you offer him/her less food in **total**, about the same, or more food than usual? | 1 = Less than usual | 2 = About the same | 3 = More than usual | 88 = NA |  |
| 1. **During** [NAME]s cough, did [NAME] Mushy (semisolid) or solid foods less than usual, the same, or more? | 1 = Less than usual | 2 = About the same | 3 = More than usual | 88 = NA |  |

| **ANXIETY AND DEPRESSION**  We would like to ask you questions about your past history and present symptoms. This information will be used to help us provide you with better medical care. However, you may find some questions upsetting. If so, please feel free not to answer. The answers to the questions will be kept confidential. Listed below are symptoms or problems that people sometimes have. Please read each one carefully and describe how much the symptoms bothered you or distressed you in the last week, including today. | | | | | | |
| --- | --- | --- | --- | --- | --- | --- |
|  | **not at all** | **a little bit** | **very much** | **extremely** | **refused** |  |
| 1. In the past week how bothered have you been by feeling suddenly scared for no reason? | 0 | 1 | 2 | 3 | 99 |  |
| 1. In the past week how bothered have you been by feeling fearful? | 0 | 1 | 2 | 3 | 99 |  |
| 1. In the past week how bothered have you been by faintness, dizziness or weakness? | 0 | 1 | 2 | 3 | 99 |  |
| 1. In the past week how bothered have you been by nervousness or shakiness inside? | 0 | 1 | 2 | 3 | 99 |  |
| 1. In the past week how bothered have you been by your heart pounding or racing? | 0 | 1 | 2 | 3 | 99 |  |
| 1. In the past week how bothered have you been by trembling? | 0 | 1 | 2 | 3 | 99 |  |
| 1. In the past week how bothered have you been by feeling tense or keyed up? | 0 | 1 | 2 | 3 | 99 |  |
| 1. In the past week how bothered have you been by headaches? | 0 | 1 | 2 | 3 | 99 |  |
| 1. In the past week how bothered were you by a spell of terror or panic? | 0 | 1 | 2 | 3 | 99 |  |
| 1. In the past week how bothered have you been by feeling like you can’t sit still or restlessness? | 0 | 1 | 2 | 3 | 99 |  |
| 1. In the past week how bothered were you by feeling low in energy, slowed down? | 0 | 1 | 2 | 3 | 99 |  |
| 1. In the past week how bothered were you by blaming yourself for things? | 0 | 1 | 2 | 3 | 99 |  |
| 1. In the past week how bothered were you by crying easily? | 0 | 1 | 2 | 3 | 99 |  |
| 1. In the past week how bothered were you by a loss of sexual interest or pleasure? | 0 | 1 | 2 | 3 | 99 |  |
| 1. In the past week how bothered were you by a poor appetite? | 0 | 1 | 2 | 3 | 99 |  |
| 1. In the past week how bothered were you by difficulty falling asleep or staying asleep? | 0 | 1 | 2 | 3 | 99 |  |
| 1. In the past week how bothered were you by feeling hopeless about the future? | 0 | 1 | 2 | 3 | 99 |  |
| 1. In the past week, have you been feeling blue? | 0 | 1 | 2 | 3 | 99 |  |
| 1. In the past week how bothered were you by feeling lonely? | 0 | 1 | 2 | 3 | 99 |  |
| 1. In the past week have you thought of ending your life? | 0 | 1 | 2 | 3 | 99 |  |
| 1. In the past week how bothered were you by feelings of being trapped or caught? | 0 | 1 | 2 | 3 | 99 |  |
| 1. In the past week how bothered were you by too much about things? | 0 | 1 | 2 | 3 | 99 |  |
| 1. In the past week, how bothered were you by feeling no interest in things? | 0 | 1 | 2 | 3 | 99 |  |
| 1. In the past week how bothered were you by feeling everything is an effort? | 0 | 1 | 2 | 3 | 99 |  |
| 1. In the past week how bothered were you by feelings of worthlessness? | 0 | 1 | 2 | 3 | 99 |  |

| **SOCIOECONOMIC STATUS** | | | | | | | |
| --- | --- | --- | --- | --- | --- | --- | --- |
|  | | | **No** | **Yes** | | **Refused** |  |
| 1. In the last three months, did you ever worry that your household would not have enough food? | | | 0 | 1 | | 99 |  |
| 1. In the last three months, did you ever reduced of balanced diet to your child because of lack of food or money to buy | | | 0 | 1 | | 99 |  |
| 1. In the last three months, did you ever reduced of balanced diet to your child because of lack of food or money to buy | | | 0 | 1 | | 99 |  |
| 1. In the last three months, did you ever reduce the number of meals for in your child a day because of shortage of food or money | | | 0 | 1 | | 99 |  |
| 1. In the last three months, did you ever reduce the number of meals of eaten child a day because of shortage of food or money | | | 0 | 1 | | 99 |  |
| 1. In the last three months, did you ever spend the whole day without eating because of shortages of food or money | | | 0 | 1 | | 99 |  |
| 1. In the last three months, did you ever ask for food or money to buy food? | | | 0 | 1 | | 99 |  |
| 1. Do you own your house? | | | 0 | 1 | | 99 |  |
| 1. Do you have land to plant or farm? | | | 0 | 1 | | 99 |  |
| 1. Does household have a bicycle? | | | 0 | 1 | | 99 |  |
| 1. Do you have radio? | | | 0 | 1 | | 99 |  |
| 1. Do you have electricity? | | | 0 | 1 | | 99 |  |
| 1. Household floor material | 1. earthen | 2. wood | | | 3.Cement/file | |  |

| **TRAUMA EVENTS**  Please indicate whether you have experienced any of the following events (check Yes or No) | | | | |
| --- | --- | --- | --- | --- |
|  | No | Yes | Refused |  |
| 1. Lack of shelter | 0 | 1 | 99 |  |
| 1. Lack of food or water | 0 | 1 | 99 |  |
| 1. Ill health without access to medical care | 0 | 1 | 99 |  |
| 1. Confiscation or destruction of personal property | 0 | 1 | 99 |  |
| 1. Combat situation (e.g. shelling or grenades) | 0 | 1 | 99 |  |
| 1. Forced evacuation under dangerous conditions | 0 | 1 | 99 |  |
| 1. Beating to the body | 0 | 1 | 99 |  |
| 1. Rape | 0 | 1 | 99 |  |
| 1. Marriage by abduction | 0 | 1 | 99 |  |
| 1. Other types of sexual abuse | 0 | 1 | 99 |  |
| 1. Knifing or axing | 0 | 1 | 99 |  |
| 1. Torture, i.e., while in captivity you received deliberate and systematic infliction of physical or mental suffering | 0 | 1 | 99 |  |
| 1. Serious physical injury from combat situation or landmine | 0 | 1 | 99 |  |
| 1. Imprisonment | 0 | 1 | 99 |  |
| 1. Forced labor (like animal or slave) | 0 | 1 | 99 |  |
| 1. Extortion or robbery | 0 | 1 | 99 |  |
| 1. Brainwashing | 0 | 1 | 99 |  |
| 1. Forced to hide | 0 | 1 | 99 |  |
| 1. Kidnapped | 0 | 1 | 99 |  |
| 1. Other forced separation from family members | 0 | 1 | 99 |  |
| 1. Forced to find and bury bodies | 0 | 1 | 99 |  |
| 1. Enforced isolation from others | 0 | 1 | 99 |  |
| 1. Someone was forced to betray you and placed you at risk of death or injury | 0 | 1 | 99 |  |
| 1. Prevented from burying someone | 0 | 1 | 99 |  |
| 1. Forced to desecrate or destroy the bodies or graves of deceased persons | 0 | 1 | 99 |  |
| 1. Forced to physically harm family member, or friend | 0 | 1 | 99 |  |
| 1. Forced to physically harm someone who is not a family member, or friend | 0 | 1 | 99 |  |
| 1. Forced to destroy someone else's property or possessions | 0 | 1 | 99 |  |
| 1. Forced to betray family member, or friend placing them at risk of death or injury | 0 | 1 | 99 |  |
| 1. Forced to betray someone who is not family or friend placing them at risk of death or injury | 0 | 1 | 99 |  |
| 1. Murder, or death due to violence, of spouse | 0 | 1 | 99 |  |
| 1. Murder, or death due to violence, of child | 0 | 1 | 99 |  |
| 1. Murder, or death due to violence, of other family member or friend | 0 | 1 | 99 |  |
| 1. Disappearance or kidnapping of spouse | 0 | 1 | 99 |  |
| 1. Disappearance or kidnapping of child | 0 | 1 | 99 |  |
| 1. Disappearance or kidnapping of other family member or friend | 0 | 1 | 99 |  |
| 1. Serious physical injury of family member or friend due to combat situation or landmine | 0 | 1 | 99 |  |
| 1. Witness beatings to head or body | 0 | 1 | 99 |  |
| 1. Witness torture | 0 | 1 | 99 |  |
| 1. Any other situation that was very frightening or felt your life was in danger. | 0 | 1 | 99 |  |
| Specifiy ------------------------------------------------------------------------------------------------------------------------------------------------------------------------------------------------------------------------------------------------------------------------------------------------------------------------------------------------------------------------------------- | | | | |

| **Pots Traumatic Stress Disorder**  The following are symptoms that people have after experiencing hurtful or terrifying events in their lives. Please read each one carefully and decide how much the symptoms bothered you **IN THE PAST WEEK** | | | | | | |
| --- | --- | --- | --- | --- | --- | --- |
|  | **Not at all** | **Little** | **Quite a bit** | **Extremely** | **Refused** |  |
| 1. Recurrent thoughts or memories of the most hurtful or terrifying events. | 0 | 1 | 2 | 3 | 99 |  |
| 1. Feeling as though the event is happening again. | 0 | 1 | 2 | 3 | 99 |  |
| 1. Recurrent nightmares. | 0 | 1 | 2 | 3 | 99 |  |
| 1. Feeling detached or withdrawn from people. | 0 | 1 | 2 | 3 | 99 |  |
| 1. Unable to feel emotions. | 0 | 1 | 2 | 3 | 99 |  |
| 1. Feeling jumpy, easily started. | 0 | 1 | 2 | 3 | 99 |  |
| 1. Difficulty concentrating. | 0 | 1 | 2 | 3 | 99 |  |
| 1. Trouble sleeping. | 0 | 1 | 2 | 3 | 99 |  |
| 1. Feeling on guard. | 0 | 1 | 2 | 3 | 99 |  |
| 1. Feeling irritable or having outburst of anger | 0 | 1 | 2 | 3 | 99 |  |
| 1. Avoiding activities that remind you of the traumatic or hurtful event. | 0 | 1 | 2 | 3 | 99 |  |
| 1. Inability to remember parts of the most traumatic or hurtful events | 0 | 1 | 2 | 3 | 99 |  |
| 1. Less interest in daily activities | 0 | 1 | 2 | 3 | 99 |  |
| 1. Feeling as if you don't have a future | 0 | 1 | 2 | 3 | 99 |  |
| 1. Avoiding thoughts or feelings associated with the traumatic or hurtful events | 0 | 1 | 2 | 3 | 99 |  |
| 1. Sudden emotional or physical reaction when reminded of the most hurtful or traumatic events | 0 | 1 | 2 | 3 | 99 |  |
| 1. Feeling that you have less skills than you had before | 0 | 1 | 2 | 3 | 99 |  |
| 1. Having difficulty dealing with new situations | 0 | 1 | 2 | 3 | 99 |  |
| 1. Feeling exhausted | 0 | 1 | 2 | 3 | 99 |  |
| 1. Bodily pain | 0 | 1 | 2 | 3 | 99 |  |
| 1. Troubled by physical problem(s) | 0 | 1 | 2 | 3 | 99 |  |
| 1. Poor memory | 0 | 1 | 2 | 3 | 99 |  |
| 1. Finding out or being told by other people that you have done something that you cannot remember | 0 | 1 | 2 | 3 | 99 |  |
| 1. Difficulty paying attention | 0 | 1 | 2 | 3 | 99 |  |
| 1. Feeling as if you are split into two people and one of you is watching what the other is doing | 0 | 1 | 2 | 3 | 99 |  |
| 1. qFeeling unable to make daily plans | 0 | 1 | 2 | 3 | 99 |  |
| 1. Blaming yourself for things that have happened | 0 | 1 | 2 | 3 | 99 |  |
| 1. Feeling guilty for having survived. | 0 | 1 | 2 | 3 | 99 |  |
| 1. Hopelessness | 0 | 1 | 2 | 3 | 99 |  |
| 1. Feeling ashamed of the hurtful or traumatic events that have happened to you | 0 | 1 | 2 | 3 | 99 |  |
| 1. Feeling that people do not understand what happened to you | 0 | 1 | 2 | 3 | 99 |  |
| 1. Feeling others are hostile to you | 0 | 1 | 2 | 3 | 99 |  |
| 1. Feeling that you have no one to rely upon | 0 | 1 | 2 | 3 | 99 |  |
| 1. Feeling that someone you trusted betrayed you | 0 | 1 | 2 | 3 | 99 |  |
| 1. Feeling humiliated by your experience | 0 | 1 | 2 | 3 | 99 |  |
| 1. Feeling no trust in others | 0 | 1 | 2 | 3 | 99 |  |
| 1. Feeling powerless to help others | 0 | 1 | 2 | 3 | 99 |  |
| 1. Spending time thinking why these events happened to you | 0 | 1 | 2 | 3 | 99 |  |
| 1. Feeling that you are the only one that suffered these events. | 0 | 1 | 2 | 3 | 99 |  |
| 1. Feeling a need for revenge. | 0 | 1 | 2 | 3 | 99 |  |

| **IMMUNIZATIONS**: (locally words for immunizations to be added) | | | | | | | | |
| --- | --- | --- | --- | --- | --- | --- | --- | --- |
| 1. Did your immunized? | 0. No 🡪162 | | | 1. Yes | | |  | |
| 1. 156. Do you have vaccination card? | 0. No | | | 1. Yes | | |  | |
|  | No | Yes |  | No | yes | DK | |  |
| 1. BCG, Polio 0 | 0 | 1 |  | 0 | 1 | 88 | |  |
| 1. DPT1, Polio1 | 0 | 1 |  | 0 | 1 | 88 | |  |
| 1. DPT2 polio2 | 0 | 1 |  | 0 | 1 | 88 | |  |
| 1. DPT3 polio3 | 0 | 1 |  | 0 | 1 | 88 | |  |
| 1. Measles | 0 | 1 |  | 0 | 1 | 88 | |  |

| **ADULT NEUROLOGICAL CONDITIONS** | | | | | |
| --- | --- | --- | --- | --- | --- |
|  | **No** | **Yes** | **Don't Know** | **Refused** |  |
| 1. Have you ever suffered a loss of consciousness from being struck in the head or in an accident | 0 | 1 | 88 | 99 |  |
| 1. Do you have numbness or loss of sensation in your hands | 0 | 1 | 88 | 99 |  |
| 1. Do you have numbness or loss of sensation in your feet | 0 | 1 | 88 | 99 |  |
| 1. Have you ever had a seizure (an episode where you lost consciousness and had uncontrolled shaking) | 0 | 1 | 88 | 99 |  |
| 1. Do you occasionally have weakness in an arm that eventually returns to normal | 0 | 1 | 88 | 99 |  |
| 1. Do you occasionally have weakness in a leg that eventually returns to normal | 0 | 1 | 88 | 99 |  |
| 1. Do you have permanent weakness in an arm or hand | 0 | 1 | 88 | 99 |  |
| 1. Do you have permanent weakness in your face | 0 | 1 | 88 | 99 |  |
| 1. Do you have permanent weakness in a leg or foot | 0 | 1 | 88 | 99 |  |
| 1. Do you have blurry vision | 0 | 1 | 88 | 99 |  |
| 1. Do you ever have temporary loss of vision | 0 | 1 | 88 | 99 |  |
| 1. Do you ever have shaking in an arm or leg even when you keep them still | 0 | 1 | 88 | 99 |  |
| 1. Do you ever have bothersome shaking in an arm or hands when you are reaching for objects | 0 | 1 | 88 | 99 |  |
| 1. Have you ever had difficulty speaking that eventually returned to normal | 0 | 1 | 88 | 99 |  |
| 1. Do you have permanent difficulties speaking | 0 | 1 | 88 | 99 |  |
| 1. Do you walk with a limp | 0 | 1 | 88 | 99 |  |
| 1. Do you have back pain on a regular basis | 0 | 1 | 88 | 99 |  |
| 1. Do you have neck pain on a regular basis | 0 | 1 | 88 | 99 |  |
| 1. Do you have pain that radiates across the face | 0 | 1 | 88 | 99 |  |
| 1. Do you have uncontrolled jerking/twitching of your face | 0 | 1 | 88 | 99 |  |
| 1. Do you ever have pain in your hands that wakes you from sleep | 0 | 1 | 88 | 99 |  |
| 1. Do you frequently loose control of your bowel or bladder | 0 | 1 | 88 | 99 |  |

| **CHILD (OLDER 2-9) NEUROLOGICAL CONDITION** | | | | | | |
| --- | --- | --- | --- | --- | --- | --- |
| 1. Is a child between 2 and 9 years of age in this household? | | | 0. No🡪197 | | 1. Yes |  |
| 1. 185. Age | | | years | | |  |
| 1. 186. Sex | | | 0 = male | 1= = female | |  |
|  | **No** | **Yes** | **Don't Know** | **Refused** | |  |
| 1. Compared with other children, did the child have any serious delay in sitting, standing or walking? | 0 | 1 | 88 | 99 | |  |
| 1. Compared with other children, does the child have difficulty in seeing, either in the daytime or at night? | 0 | 1 | 88 | 99 | |  |
| 1. Does the child appear to have difficulty in hearing? | 0 | 1 | 88 | 99 | |  |
| 1. When you tell the child to do something, does he/she seem to understand what you are saying? | 0 | 1 | 88 | 99 | |  |
| 1. Does the child have difficulty in walking or moving his/her arms or does he/she have weakness and/or stiffness in the arms or legs? | 0 | 1 | 88 | 99 | |  |
| 1. Does the child sometimes have fits, become rigid, or lose consciousness? | 0 | 1 | 88 | 99 | |  |
| 1. Does the child learn to do things like other children of his/her age? | 0 | 1 | 88 | 99 | |  |
| 1. Does the child speak at all (can he/she make himself/herself understood in words; can he/she say some recognizable words)? | 0 | 1 | 88 | 99 | |  |
| 1. Is the child’s speech in any way different from normal (not clear enough to be understood by people other than his/her immediate family)? | 0 | 1 | 88 | 99 | |  |
| 1. Compared with other children of his/her age, does the child appear in any way mentally backward, dull or slow? | 0 | 1 | 88 | 99 | |  |

199. **Think of this ladder as representing where people stand in your kebele**

At the top of the ladder are people who very very rich- those who have most money and household assets. At the bottom are those who are very very poor- those who have least money and household assets. The higher up you are in this ladder, the closer you are to the people at the top. The lower down you are, the closer you are to the people the very bottom.


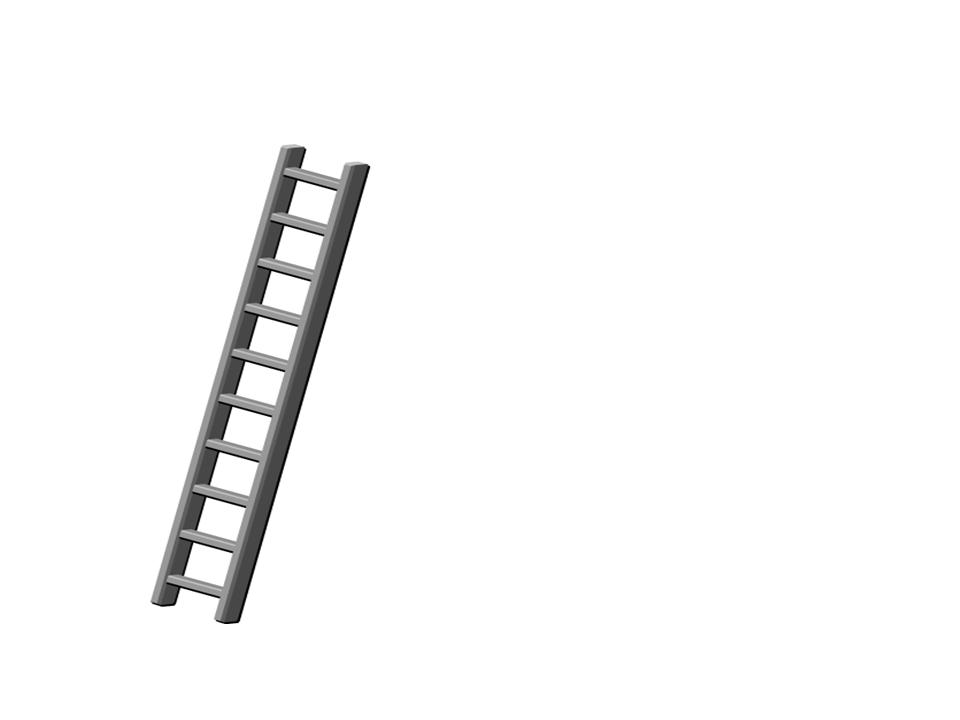
Please put an X mark on the rung you think and you stand at this time in your life, relative to other people in your kebele or community. Write the number on in the blank space

1^st^— very very poor

10th rank– Very very reach

1^st^— very very poor

| **ANTHROPOMETRY** | | | | |
| --- | --- | --- | --- | --- |
| 197. Child | Weight |  | length |  |
| 198 Father | weight |  | Height |  |

Thank you!!

Interview indeed (HH:MM) ---------------------------------------
