# Supplementary material for: Household food insecurity and symptoms of neurologic disorder in Ethiopia: An observational analysis
Source: BMC Public Health. 2010 Dec 31;10:802. doi: 10.1186/1471-2458-10-802 (PMC3027184; doi:10.1186/1471-2458-10-802)
Supplement: Additional file 2 — Survey questionnaire for women and children in Amharic. The survey questionnaire used to obtain data about women in the analysis in Amharic. [file 1471-2458-10-802-S2.PDF]

ሚስጥራዊነቱ የተጠበቀና ለጥናት ብቻ የሚውል መረጃ

የመረጃ ሰብሳቢው ስም		የቀበሌው ስም -------------------	የቤተሰብ መለያ ቁጥር:-----------
የተቆጣጣሪው ስም		የቤት ቁጥር --------------------	የእናት መለያ ቁጥር-------------
የተቆጣጣሪው ፊርማ:			የህፃኑ/ኗ መለያ ቁጥር-------------
የመጀመሪያው ጉብኝት ውጤት	1 ተጠናቋል 2 በቤት የሉም<br>3 ፍቃደኛ አልሆኑም 4 ተቋርጧል		ተለዋጭ የቃለመጠይቅ ቀጠሮ የተያዘበት ቀን:
የሁለተኛው ጉብኝት ውጤት	1 ተጠናቋል 2 በቤት የሉም<br>3 ፍቃደኛ አልሆኑም 4 ተቋርጧል		ተለዋጭ የቃለመጠይቅ ቀጠሮ የተያዘበት ቀን:
የሦስተኛው ጉብኝት ውጤት	1 ተጠናቋል 2 በቤት የሉም<br>3 ፍቃደኛ አልሆኑም 4 ተቋርጧል		ተለዋጭ የቃለመጠይቅ ቀጠሮ የተያዘበት ቀን:
ቃለ መጠይቁ የተካሄደበት ቀን/ወር/ ዓም	-------- /--------/-----------	መጠይቁ የተጀመረበት ሰዓት:	
የህፃን ጾታ		0 ፡ ወንድ<br>1 ፡ ሴት	
ህፃኑ/ኗ በሕይወት አለ		0 ፡ የለም<br>1 ፡ አለ	

እድሜና የትምህርት ደረጃ	
1.እድሜዎ ስንት ነው	------------------- አመታት
2. ትምህርት ለስንት አመታቶች ያሕል ተምረዋል	------------------- አመታት

ክፍል ሀ፡ ዕድሜን ያገናዘበ የህፃኑ/ኗ አጠቃላይ ዕድገትን በተመለከተ	
ስለ ልጅዎ አንዳንድ ጥያቄዎችን መጠየቅ እጀምራለሁ:: በአሁኑ ጊዜ ልጅዎ ቀጥሎ የምዘረዝርልዎትን ድርጊቶች ያለምንም አገዛ በራሱ/ሷ ማድረግ መቻል አለመቻሉ/ሏ/ን እንዲነግሩኝ እፈልጋለሁ::	
3. የልጅዎ የትውልድ ቀን መቼ ነበር /ወሩም ጭምር ይመዝገብ/	-------- /--------/-----------<br>ቀን ወር ዓም
4. የልጅዎ ዕድሜ በወራት ሲሰላ ስንት ነዉ	------------------- ወር

ሶሳል	አዎ	የለም		አይቻለሁ	አላየሁም	
የሌሎችን ፈገግታ ተከትሎ/ላ ፈገግ ይላል/ትላለች				2	3	
በራሱ/ሷ ጊዜ ፈገግ ይላል/ትላለች	1	0		2		
መጫዎቻ ለማግኘት ይሞክራል				2	3	
እጁ/ጇን በማወዛወዝ ደህና ሁኑ ይላል	1	0		2		
ከፈታኝ ሰው ጋር ኳስ ይጫወታል/ትጫወታለች	1	0		2		

በስኒ/ኩባያ... መጠጣት ይችላል/ትችላለች				2	3	
አንዳንድ ድርጊቶችን ሌሎች እንደሚያደርጉት ለማድረግ ይሞክራል/ትሞክራለች	1	0		2		
በቤት ውስጥ ለማገዝ ይሞክራል/ትሞክራለች	1	0		2		
ልብሱን/ሷን ማውለቅ ይችላል/ትችላለች	1	0		2		
መልበስ ይችላል/ትችላለች	1	0		2		
እጁን/ጇን መታጠብ ይችላል/ትችላለች	1	0		2		
የጎደኛን ስም መጥራት ይችላል/ትችላች	1	0		2		
ልብስ ያለእርዳታ መልበስ ይቸላል/ትችላች	1	0		2		

ፋይን ሞተር	አዎ	የለም		አይቻለሁ	አላየሁም	
የሚቀሳቀሱ ነገሮችን ተከትሎ ከመሃል አለፍ ብሎ ይከተላል				2	3	
የሚንቀሳቀሱ ነገሮችን ተከትሎ ከቀኝ ወደ ግራ ወይም ከግራ ወደ ቀኝ 180 ዲግሪ ይከተላል/ትከታተላለች				2	3	
የሚንኮሻኮሽ/የሚንቀጫቀጩ መጫወቻ/ዕቃዎችን ይይዛል/ትይዛለች				2	3	
ዕቃዎችን ለመያዝ ይጣጣራል/ትጣጣራለች/ትሞክራለች				2	3	
ትናንሽ ዕቃዎችን በእጁ/ጇ አጥብቆ/ቃ /ጨምድዶ/ዳ/ ይይዛል/ትይዛለች				2	3	
ሁለት ኩቦችን ያጋጫል				2	3	
ጡቦችን በመጠጫ ዕቃዎች ውስጥ መክተት ይችላል/ትችላለች				2	3	
2 ጡቦችን ደራርቦ/ባ ማስቀመጥ ይችላል/ትችላለች				2	3	
4 ጡቦችን ደራርቦ/ባ ማስቀመጥ ይችላል/ትችላለች				2	3	
6 ጡቦችን ደራርቦ ማስቀመጥ ይችላል/ትችላለች				2	3	
8 ጡቦችን ደራርቦ ማስቀመጥ ይችላል/ትችላለች				2	3	

ፊ	አዎ	የለም		አይቻለሁ	አላየሁም	
የደወል/ሌላ ድምፅ/ በሰማ/ች ጊዜ ወደ ድምፁ አቅጣጫ ይመለከታል/ትመለከታለች ወይም ዓይኑን ያንቀሳቅሳል				2	3	
ድምፅ ለማውጣት ይጣጣራል/ትጣጣራለች	1	0		2		
ቀጭን ድምፅ ያሰማል (የደስታ/የጫወታ)	1	0		2		
ይስቃል/ለች/	1	0		2		
የሌሎች ሰዎችን የንግግር ድምጽ ለመድገም ይሞክራል/ትሞክራለች	1	0		2		
ማማ እና ባባን የሚሉትን ቃላት ሳይለይ ይጠቀማል	1	0		2		
ለይቶ/ታ ማማ እና ባባ ይላል/ትላለች/	1	0		2		
እስከ 2 ቃላት መናገር ይችላል/ትችላለች	1	0		2		
እስከ 3 ቃላት መናገር ይችላል/ትችላለች	1	0		2		
እስከ 6 ቃላት መናገር ይችላል/ትችላለች	1	0		2		
ለስዕል ስያሜ ይሰጣል/ትሰጣለች				2	3	

6 የሰውነት ክፍሎችን ለይቶ/ታ መጠቆም ይችላል/ትችላለች /ጆሮ፣ አፍንጫ፣ ዓይን፣ አፍ፣ እጅ እና እግር/				2	3	
እስከ አራት የሚደርሱ የተለያዩ ስዕሎችን ለይቶ/ታ መጠቆም ይችላል/ትችላለች /ሰው፣ ዶሮ፣ በሬ እና በግ/				2	3	
በከፊል ንግግሩ/ሯን ይረዱታል፡ መረዳት የሚቻል ንግግር ይናገራል/ትናገራለች				2	3	
ሙሉ በሙሉ መናገር የሚችል/የምትችል				2	3	
አራት የቀለም አይነት መጥራት ይችላል/ትችላለች				2	3	
የኩባያን ጥቅም ቢጠየቅ/ብትጠየቅ መመለስ ይችላል/ትችላለች				2	3	
የወንበርን ጥቅም ቢጠየቅ/ብትጠየቅ መመለስ ይችላል/ትችላለች				2	3	

ገሮስ ሞተር	አዎ	የለም		አይቻለሁ	አላየሁም	
በደረቱ/ቷ ከተኛበት/ችበት ጭንቅላቱን/ቷን ያነሳል/ታነሳለች				2	3	
ቀና ይላል/ትላለች ጭንቅላቱን/ቷ/ን በ45 ዲግሪ በደረቱ/ቷ ከተኛበት/ችበት ቀና ያደርጋል/ታደርጋለች/				2	3	
ጭንቅላቱ/ቷ/ን በ90 ዲግሪ በደረቱ/ቷ ከተኛበት/ችበት ቀና ያደርጋል/ታደርጋለች/ ያንጥጣል/ታንጋጥጣለች/				2	3	
ለመቀመጥ ይጣጣራል				2	3	
ያለ ድጋፍ መቀመጥ ይችላል				2	3	
ዕቃዎችን በመደገፍ ይቆማል/ትቆማለች				2	3	
ለመቆም ይፍጨረጨራል/ትፍጨረጨራለች				2	3	
ያለምንም ድጋፍ በራሱ/ሷ ጊዜ ለ2 ሴኮንዶች ያህል ይቆማል/ትቆማለች				2	3	
ያለምንም ድጋፍ ይቆማል				2	3	
መራመድ ይችላል/ትችላለች				2	3	
ወደኃላ መራመድ ይችላል/ትችላለች				2	3	
መሮጥ ይችላል/ትችላለች				2	3	
ኳስን ወደፊት መምታት/መለጋት ይችላል/ትችላለች				2	3	
ደረጃዎችን ይወጣል/ትወጣለች				2	3	
ኳስን ከጭንቅላቱ/ቷ በላይ ወደፊት ይወረውራል/ትወረውራለች				2	3	
ሁለት እግሮችን አንስቶ መዝለል ይችላል				2	3	
በአንድ እግሩ/እግርዋ ከ 1 ሴኮንድ በላይ መቆም ይችላል/ትችላለች				2	3	
በአንድ እግሩ/እግርዋ ከ 2 ሴኮንድ በላይ መቆም ይችላል/ትችላለች				2	3	
ሁለቱንም እግሮችን ወደ ፊት እንጣጥ ማለት ይችላል/ትችላለች				2	3	

ክፍል ሰ፡ ለህፃኑ የሚደረግ እንክብካቤን በተመለከተ

አሁን ደግሞ ስለ ልጅዎ አመጋገብ አንዳንድ ጥያቄዎችን እጠይቀዎታለሁ፡፡

	የለም	አዎ	
1. (ስም)ን ጡት አጥብተው ያውቃሉ	0 → 5	1	
2. (ስም)ን ጡት ማጥባት የሚጀምሩት ከወለዱ ከስንት ጊዜ በኃላ ነዉ	ደቂቃ		
	ሰዓታት		
	ቀናት		

	የለም	አዎ	
3. ለ (ስም) ጡት ከማስጀመርዎ በፊት የሰጡት ፈሳሽ ወይም ሌላ ምግብ /ለምሳሌ፡ቅቤ/ ነበር	0	1	
4. ከትናንት በዚህ ሰዓት ጀምሮ እስከ አሁን ባለው ጊዜ (ስም)ን ጡት አጥብተዋታል/ታል	0	1	
ከሚከተሉት የትኛቹን ለ(ስም) ተሰጥቶት/ቷት ያውቃል	የለም	አዎ	
5. ቫይታሚን ወይም ተጨማሪ ንጥረ-ነገሮች፡መድሃኒቶች	0	1	
6. የመጠጥ ውሃ	0	1	
7. ጣፋጭ ነገር የተደባለቀበት ውሃ	0	1	
8. ጭማቂ ወይም ሻይ	0	1	
9. የተቀመመ/የተዘጋጀ/ የህፃናት ምግብ	0	1	
10. የታሸገ የዱቄት ወተት ወይም የእንስሳ ትኩስ ወተት	0	1	
11. እጥሚት	0	1	
12. ፈሳሽ ያልሆኑ ምግቦች /ለምሳሌ፡- ንፍሮ፣ገንፎ፣ ድንች.../	0	1	
13. ህይወት አድን ንጥረ ነገር /ኦ አር ኤስ/ ወይም ለምለም	0	1	
14. ማንኛውንም ዓይነት ሥጋ ወይም ዓሣ	0	1	
15. ባለፉት 2 ሳምንታት ውስጥ (ስም) ተቅማጥ ነበረው/ነበራት	0 አልነበረውም→21	1.አዎ	

(ስም) ተቅማጥ በያዘው ወቅት ከሚከተሉት የትኞቹ ነገሮች ተደርገውለ/ላ/ታል					
16. የእናት ጡት			0 አልተሰጠም	1.ተሰጥቷል	
17. ሌሎች ፈሳሾች			0 አልተሰጠም	1.ተሰጥቷል	
18. ፈሳሽ ያልሆኑ ምግቦች			0 አልተሰጠም	1.ተሰጥቷል	
19. በተቅማጡ ወቅት (ስም) የጠባው/ችው የእናት ጡት ከሌላው ጊዜ ጋር ሲነፃፀር ምን ያህል ነበር	1.ያነሰ ነበር	2.እኩል ነበር	3.የበለጠ ነበር	88.አላውቅም	
20. በተቅማጡ ወቅት (ስም) የጠባው/ችው ሌሎች ፈሳሾች ከሌላው ጊዜ ጋር ሲነፃፀር ምን ያህል ነበር	1.ያነሰ ነበር	2.እኩል ነበር	3.የበለጠ ነበር	88.አላውቅም	
21. በተቅማጡ ወቅት ለ(ስም) ይሰጥ የነበረው ምግብ ከሌላው ጊዜ ሲነፃፀር ምን ያህል ነበር	1.ያነሰ ነበር	2.እኩል ነበር	3.የበለጠ ነበር	88.አላውቅም	
22. ባለፉት ሁለት ሳምንታት ወስጥ (ስም) ትኩሳት ነበረው/ራት		0.አልነበረውም→ 27	1.አዎ	99 ፍቃደኛ አይደሉም	

በትኩሳቱ ወቅት ከሚከተሉት የትኛቹ ለ(ስም) ተሰጥተዋል					
23. የእናት ጡት		0.አልተሰጠም	1.አዎ	99	
24. ሌሎች ፈሳሾች		0.አልተሰጠም	1.አዎ	99	
25. ፈሳሽ ያልሆኑ ምግቦች		0.አልተሰጠም	1.አዎ	99	
26. በትኩሳቱ ወቅት (ስም) የጠባው/ችው ጡት ከሌላው ጊዜ ጋር ሲነፃፀር ምን ያህል ነበር?	1. ያነሰ ነበር	2.እኩል ነበር	3. ይበልጥ ነበር	88.አላውቅም	
27. በትኩሳቱ ወቅት (ስም) የተሰጣት/የተሰጠው ሌሎች ፈሳሾች ከሌላው ጊዜ ጋር ሲነፃፀር ምን ያህልነበር?	1. ያነሰ ነበር	2.እኩል ነበር	3. ይበልጥ ነበር	88.አላውቅም	

28. በትኩሳቱ ጊዜ ለ(ስም) የተሰጣት/የተሰጠው አጠቃላይ ምግብ ከሌላው ጊዜ ሲነፃፀር ምን ያህል ነበር	1.ከሌላው ጊዜ ያነሰ ነበር	2.እኩል ነበር	3.ከሌላው ጊዜ ይበልጥ ነበር	88.አላውቅም	
28. (ስም) ባለፉት 2 ሣምንታት ውስጥ ያስላት/ለው ነበር			0.አልሳለም/ችም→33	1.አዎ	
ለ(ስም) በሳሉ/ሏ ወቅት ከሚከተሉት የትኛቹን ነገሮች ተሰጥተውታል/ዋታል					
30. የእናት ጡት			0.አልተሰጠውም/ትም	1.አዎ	
31. ሌሎች ፈሳሾች			0.አልተሰጠውም/ትም	1.አዎ	
32. ፈሳሽ ያልሆኑ ምግቦች			0.አልተሰጠውም/ትም	1.አዎ	
33. በሣሉ ወቅት (ስም) ጊዜ ያጠቡት/ቧት ጡት ከሰላው ጊዜ ሲነፃፀር ምን ያህል ነበር	1.ያነሰ ነበር	2.እኩል ነበር	3.የበለጠ ነበር	88.አላውቅም	
34. በሣሉ ወቅት (ስም) ጊዜ የነበረው ሌሎች ፈሳሾች ከሰላው ጊዜ ሲነፃፀር ምን ያህል ነበር	1.ያነሰ ነበር	2.እኩል ነበር	3.የበለጠ ነበር	88.አላውቅም	
35. በሣሉ ወቅት (ስም) ጊዜ ይሰጠው/ጣት የነበረው ምግብ ከወትሮው ሲነፃፀር፡- ምን ያህል ነበር	1.ያነሰ ነበር	2.እኩል ነበር	3.የበለጠ ነበር	88.አላውቅም	

USE OF SERVICES
የአገልግሎቶችን አጠቃቀም በተመለከተ

አሁን ስለ እርግዝናዎ የሚያመላክቱ ጥያቄዎች መጠየቅ እጀምራለሁ፡፡

36. ባለፉት አምስት አመታት ውስጥ ስንት ጊዜ አርግዘው ያውቃሉ ?	______እርግዝና
37. ባለፉት አምስት አመታት ውስጥ ስንተ ልጆች ወልደዋል ?	______ልጆች
38. የመጨረሻ ልጅዎት በስንት ዓመተ ምህረት ነው የተወለደው/የተወለደችው ?	______ዓመተ ምህረት
39. በጠቅላላ ስንት ልጆች አሉዎት ?	______ ልጆች
40. ከልጆችሽ ውስጥ ስንቶቹ በጤና ጣብያ ተወልደዋል (ለምሳሌ በጤና ጣበያ፡ በጤና ማዕከል ፡ በሆስፒታል)	______ ልጆች

	የለም	አዎ	ፍቃደኛ አይደሉም	
41. ሞቶ የተወለድ ልጅ ኖሮዎት ያውቃል ወይ?	0	1	99	
42. በተወለደ/በተወለደች በአንድ ወሩ ውስጥ የሞቱ ልጆች ነበሮት?	0	1	99	
43. የጤና ህክምና ዋስትና አሉት ?	0	1	99	

These questions are about the health care facilities nearest you

ከዚህ በታች ያሉት ጥያቄዎች ባካባበዎ ስላሉ የህክምና ጣብዮች የሚመለከቱ ናቸው

	እጅግ በጣም ጥሩ	በጣም ጥሩ	ጥሩ	መደበኛ	የደከመ	ፍቃደኛ አይደሉም

44. ለእርሶ የሚቀርቦት የጤና ጣቢያ በየት ይገኛል?	____________ሰፍራ/ቦታ	
45. ከ 0 እስከ 99 ባለው መለክያ፣ ጤና ጣቢያው ምን ያህል የአገልግሎት ጥራት አለው ብለው ይገምታሉ?	0 እጅግ በጣመ ጥሩ<br>1 በጣም ጥሩ<br>2 ጥሩ<br>3 መደበኛ<br>4 የደከመ<br>99 ፍቃደኛ አይደሉም	
46. ለእርሶ የሚቀርቦት የጤና ማዕከል በየት ይገኛል?	____________ ሰፍራ/ቦታ	
47.ከ 0 እስከ 99 ባለው መለክያ፣ ጤና ማዕከሉ ምን ያህል የአገልግሎት ጥራት አለው ብለው ይገምታሉ?	0 እጅግ በጣመ ጥሩ<br>1 በጣም ጥሩ<br>2 ጥሩ<br>3 መደበኛ<br>4 የደከመ<br>99 ፍቃደኛ አይደሉም	
48. ለእርሶ የሚቀርቦት ሆስፒታል በየት ይገኛል?	____________ ሰፍራ/ቦታ	
49. ከ 0 እስከ 99 ባለው መለክያ፣ሆስፒታሉ ምን ያህል የአገልግሎት ጥራት አለው ብለው ይገምታሉ?	0 እጅግ በጣመ ጥሩ<br>1 በጣም ጥሩ<br>2 ጥሩ<br>3 መደበኛ<br>4 የደከመ<br>99 ፍቃደኛ አይደሉም	
50. ከ 0 እስከ 99 ባለው መለክያ፣ ዛሬ ጤናዎትን በምን ያክል መጠን ያዩታል??	0 እጅግ በጣመ ጥሩ<br>1 በጣም ጥሩ<br>2 ጥሩ<br>3 መደበኛ<br>4 የደከመ<br>99 ፍቃደኛ አይደሉም	

**These questions are about your last pregnancy**<br>**ስለመጨሻ እርግዝናዎ በተመለከተ**		
51. ባለፈው ባረገዙ ጊዘ፣ ስንት ጊዜ ወደ ጤና ባለሙያ ጋር ለመታየት ሄደው ያውቀሉ?	1. ሄጄ አላውቅም<br>2. አንዴ<br>3. ሁለት ጊዜ<br>4. ሦስት ጊዜ<br>5. ከሦስት ጊዜ በላይ<br>99.አላውቅም	
መልሱ የለም ከሆነ ወደ ጥያቄ ቁጥር 55 ያምሩ		
52. በነፍስ ጡር በሆኑ ጊዜ የጤና አገልግሎት የት ነው ያገኙት?	1 ስም (ቦታ)___________________<br>2 እቤት<br>3 አዋላጅ ቤት<br>99 አላቅም	
53. የመጨረሻ ልጅሽን ስትውልጂ የት ቦታ ነው የወለድሽው	1 ቤት<br>2 ሌላ ሰው ቤት/አዋላጅ ቤት<br>3 የመንግስት ጤና ጣቢያ<br>4 የመንግስት ጤና ማዕከል<br>5 የመንግስት ሆስፒታል<br>6 የሚሽን ጤና ጣብያ<br>7 የሚሽን ጤና ማዕከል<br>8 የሚሽን ሆስፒታል<br>9 የግል ጤና ጣብያ<br>10 የግል ጤና ማዕከል<br>11 የግል ሆስፒታል<br>12 ሌላ(ዝርዝር)<br>99 አላቅም	
54. ከተዘረዘሩት ውስጥ የትኛው ቦታ ነው??<br>የቦታ ሥም	የቦታ ሥም<br>99 አላቅም	

ክፍል ሐ፡ ጭንቀትና ድብርትን በተመለከተ

ስላለፈው ህይወትዎ እና በአሁን ሰዓት ስላሉት አንዳንድ የጭንቀትና ድብርት ምልክቶች ልንጠይቅዎ እንፈልጋለን፡፡ እርስዎ በግልፅነት የሚሰጡን መረጃ ተገቢ የሆነ የህክምና አገዛ እንዲደረግልዎት ይረዳናል፡፡ ለጥያቄዎቹ የሚሰጡን መልስ ሚስጥራዊነቱ የተጠበቀ ነው፡፡ ቀጥሎ የተዘረዘሩት የጭንቀት ወይም የድብርት ምልክቶች በብዙ ሰዎች ዘንድ አንዳንዴ የተለመዱ ናቸው፡፡ እያንዳንዱን ጥያቄ በጥንቃቄ ካዳመጡ በኃላ ላለፈው ሳምንት /ዛሬን ጨምሮ/ ምን ያህል እርሶን እንዳወከዎት ይግለፁ፡

	በፍፁም	በመጠኑ	በከፍተኛ	በጣም ከፍተኛ	ፍቃደኛ አይደሉም	
55. ባለፈው ሳምንት ያለምንም ምክንያት ድንገት ውስጥዎን ፍርሃት ፍርሃት ይለው ነበር	0	1	2	3	99	
56. ባለፈው ሳምንት በፍርሃት ስሜት የተነሳ ምን ያህል አስበዎል	0	1	2	3	99	
57. ባለፈው ሳምንት የድካም፡ የመዝለፍለፍ ወይም ራስዎን የመክበድ ስሜትዎ ምን ያህል አሳስቦታል	0	1	2	3	99	
58. ባለፈው ሳምንት የመረበሽ ወይም የመፍራት ስሜትዎስ ምን ያህል አሳስብዎት ነበር	0	1	2	3	99	
59. ባለፈው ሳምንት ልብዎ በሀይል ወይም ቶሎ ቶሎ መምታት ምን ያህል አሳስብዎት ነበር	0	1	2	3	99	
60. ባለፈው ሳምንት ሰውነትዎ በፍርሃት ይንቀጠቀጥብዎ ነበር	0	1	2	3	99	
61. ባለፈው ሳምንት የሰውነትዎ መጨናነቅ ስሜትዎ ምን ያህል አሳስብዎት ነበር	0	1	2	3	99	
62. ባለፈው ሳምንት የራስ ምታት መኖር ምን ያህል አሳስብዎት ነበር	0	1	2	3	99	
63. ባለፈው ሳምንት በአጋጣም መሸበር ወይም ከፍተኛ ፍርሓት ስሜትዎስ ምን ያህል አሳስብዎት ነበር	0	1	2	3	99	
64. ባለፈው ሳምንት ተረጋግቶ ያለመቀመጥ ወይም ዕረፍት የማጣት ስሜትዎስ ምን ያህል አሳስብዎት ነበር	0	1	2	3	99	
	በፍፁም	በመጠኑ	በከፍተኛ	በጣም ከፍተኛ	ፍቃደኛ አይደሉም	
65. ባለፈው ሳምንት ብርታት/አቅም የማጣት ስሜትዎስ ምን ያህል አሳስብዎት ነበር	0	1	2	3	99	
66. ባለፈው ሳምንት በሆነ ባልሆነው ራስዎን ይወቅሱ ነበር	0	1	2	3	99	
67. ባለፈው ሳምንት በቀላሉ አልቅስ/ሽ አልቅስ/ሽ የሚል ስሜት ምን ያህል አሳስብዎት ነበር	0	1	2	3	99	
68. ባለፈው ሳምንት የፍትዎት ስሜት ማጣት ምን ያህል አሳስብዎት ነበር	0	1	2	3	99	
69. ባለፈው ሳምንት የምግብ ፍላጐት ማጣትስ ምን ያህል አሳስብዎት ነበር	0	1	2	3	99	
70. ባለፈው ሳምንት የእንቅልፍ ማጣት ችግር ምን ያህል አሳስብዎት ነበር	0	1	2	3	99	

71. ባለፈው ሳምንት የተስፋ መቁረጥ ስሜትዎስ ምን ያህል አሳስብዎት ነበር	0	1	2	3	99	
72. ባለፈው ሳምንት አዝነው ወይም ተክዘው ነበር	0	1	2	3	99	
73. ባለፈው ሳምንት የብቸኝነት ስሜትስ ምን ያህል አሳስብዎት ነበር	0	1	2	3	99	
74. ባለፈው ሳምንት ህይወትዎን የማጥፋት ሀሣብ ይመጣብዎ ነበር	0	1	2	3	99	
75. ባለፈው ሳምንት በስራም ይሁን በሌሳ ጉዳይ የመጠመድ ስሜት ምን ያህል አሳስብዎት ነበር	0	1	2	3	99	
76. ባለፈው ሳምንት ስነገሮች አብዝተው ይጨነቁ ነበር	0	1	2	3	99	
77. ባለፈው ሳምንት ስነገሮች ሁሉ ስምንም ዓይነት ፍላጐት ማጣት ስሜት ምን ያህል አሳስብዎት ነበር	0	1	2	3	99	
78. ማንኛው ነገር የሚገኘው ወይም የሚሳካው በድካም ነው እያሉ ምን ያህል ተጨንቋዋል	0	1	2	3	99	
79. ባለፈው ሳምንት የዋጋ-ቢስነት ስሜትስ ምን ያህል አሳስብዎት ነበር	0	1	2	3	99	

ክፍል መ፡ ማህበራዊና ኢኮኖሚያዊ ሁኔታን በተመለከተ				
	የለም	አዎ	ፍቃደኛ አይደሉም	
80.ባለፉት ሦስት ወራት ውስጥ ቤትዎ ውስጥ በቂ ምግብ አይኖርም ብለው ተጨንቀው ያውቀሉ ወይ?	0	1	99	
81. ባለፉት ሦስት ወራት ውስጥ እርሶ ወይንም ቤተሰብዎ ተጨማሪ የምግብ መግዣ ገንዘብ ሳይኖሮት ወይም ሳይኖራችሁ ምግብ ያለቀበት ጊዜ ነበረ ወይ?	0	1	99	
82. ባለፉት ሦስት ወራት ውስጥ በምግብ ወይንም በገንዘብ እጥረት ምክናያት የሚበሉትን የምግቦች አይነት ቁጥር ቀንሰው ያውቃሉ ወይ?	0	1	99	
83. ባለፉት ሦስት ወራት ውስጥ ልጆችዎ በምግብ ወይንም በገንዘብ እጥረት ምክንያት በቂ ምግብ ያልበሉበት ጊዜ ነበረ ወይ?	0	1	99	
84. ባለፉት ሦስት ወራት ውስጥ በገንዘብ ወይም በምግብ እጥረት ምክንያት እርሶ ወይንም ቤትዎ ውስጥ ያሉ በቀን የሚበሉት ጊዜዎች ቀንሰው ያውቃሉ ወይ?	0	1	99	
85. ባለፉት ሦስት ወራት ውስጥ በገንዘብ ወይም በምግብ እጥረት ምክንያት ሙሉ ቀን ያልበሉበትጊዜ ነበረ ወይ?	0	1	99	
86. ባለፉት ሦስት ወራት ውስጥ ምግብ ለመግዛት ገንዘብ ወይንም ምግብ ጠይቀው ያውቀሉ ወይ?	0	1	99	
87. የግልዎ የሆነ ቤት አለዎት	0	1	99	
88. ለእርሻ ወይም አትክልት የሚሆን የግል ይዞታ መሬት አለዎት	0	1	99	
89. በቤትዎ ብስክሌት አለ	0	1	99	
90. ራዲዩስ አለ	0	1	99	

91. ቤታችሁ ኤሌክትሪክ አለዉ		0	1	99
92. የቤታችሁ ወለል ምንድን ነዉ	1. አፈር	2. ጣውላ		3.ልሾ/ሲሚንቶ

ክፍል ሠ: አስጨናቂ ገጠመኞችን በተመለከተ፤ ከዚህ በፊት ቃለ መጠይቅ ለተደረጉ ባለፈው 6 ወር ውስጥ

የሚከተሉት ሁኔታዎች ተከስተው እንደሆነ ይጠይቁ ከዚህ በፊት ቃለ መጠይቅ ላልተደረጉ በሂወት ዘመናቸው የሚከተሉት ሁኔታዎች አጋጥማዋቸው እንደሆነ ይጠይቁ

	የለም	አዎ	ፍቃደኛ አይደሉም	
93. መጠለያ ማጣት	0	1	99	
94. ምግብ ወይም ውሃ ማጣት	0	1	99	
95. ምንም ዓይነት ህክምና ያልተደረገለት ህመም ነበረብዎ	0	1	99	
96. የግል ንብረትዎ መውደም ወይም መወረስ	0	1	99	
97. በጦርነት ወይም ውጊያ ውስጥ መሳተፍ/በተለይም በቦምብና መድፍ የታገዘ/	0	1	99	
98. በአደገኛ ሁኔታ አካባቢውን እንዲለቁ መገደድ	0	1	99	
99. ተደባድበው ያውቃሉ	0	1	99	
100. ተገደው ተደፍረውስ ያውቃሉ	0	1	99	
101. የጠለፋ ጋብቻ ተፈጽሞብዎታል	0	1	99	
102. የተለያዩ ፆታዊ ጥቃቶችስ ደርሶቦት ያውቃል	0	1	99	
103. በጩቤ መወጋት ወይም በለሳ ለለት /ለምሳሌ ገጀራ/ መቆረጥስ	0	1	99	
104. በእስር ወይም ምርኮ የተነሳ አካላዊና አዕምሮአዊ ስቃይ ደርሰቦዎታል	0	1	99	
105. በጦርነት ወይም በፍንዳታ የተነሳ ከባድ የአካል ጉዳትስ ደርቦብዎታል	0	1	99	
106. እስር ቤት ታስረው ያውቃሉ	0	1	99	

	የለም	አዎ	ፍቃደኛ አይደሉም	
107. የግዳጅ ስራ /ልክ እንደ እንስሳ ወይም እንደ ባሪያ/ እንዲሰሩ ተደርገው ያውቃሉ	0	1	99	
108. በግዳጅ ወይም በስርቆት ገንዘብዎ ወይም ንብረትዎ ተዘርፎ ያውቃል	0	1	99	
109. አንድን ፅንሰ-ሃሳብ እንዲቀበሉት በሌሎች ግለሰቦች በተደጋጋሚ ተሰብከው ያውቃሉ	0	1	99	
110. ለመሸሸ ሲሉ ተደብቀው ያውቃሉ	0	1	99	
111. መታገት ወይም ታፍኖ መወሰድስ	0	1	99	
112. ከቤተሰብዎ አባላት ተገዶ የመለየት አጋጣሚስ ነበረብዎ	0	1	99	
113. ሬሳን ፈልገው እንዲቀብሩ ተገደው ያውቃሉ	0	1	99	
114. ከሌሎች ሰዎች እንዲለዩ ተገደው ያውቃሉ	0	1	99	
115. ሌላ ሰው እንዲክድዎትና ለሞት ወይም የማቁሰል አደጋ እንዲያጋልጥዎት ተገዶ ያውቃል	0	1	99	
116. የሞተበዎትን ሰው እንዳይቀብሩ ተከልክለው ያውቃሉ	0	1	99	
117. የሙታንን ቅሬት ወይም መካነ-መቃብር እንዲያወድሙ ተገደው ያውቃሉ	0	1	99	
118. በቤተሰብዎ አባል ወይም ጓደኛዎ ላይ አካላዊ ጉዳት እንዲያደርሱ ተገደው ያውቃሉ	0	1	99	

119. ዘመድ ወይም ጓደኛ ባልሆነ ሰወስ ሳዪ አካሳዊ ጉዳት እንዲያደርሱ ተገደው ያውቃሉ	0	1	99	
120. የሌሳ ሰውን ንብረት ወይም ይዞታ እንዲያወድሙ ወይም እንዲያጠፉ ተገደው ያውቃሉ	0	1	99	
121. የቤተሰብዎ አባል ወይም ጓደኛዎን እንዲከዱ ብሉም ለሞት ወይም ለአካሳዊ ጉዳት እንዲያጋልጡት ተገደው ያውቃሉ	0	1	99	
122. በሌሳ ሰውስ ሳይ (የቤተሰብዎ አባል ወይም ጓደኛዎ ያልሆነ ሰው) እንዲከዱ ብሉም ለሞት ወይም ለአካሳዊ ጉዳት እንዲያጋልጡት ተገደው ያውቃሉ	0	1	99	
123. በግጭት ወይም በረብሻ የትዳር አጋርዎ ተገድሎቦታል	0	1	99	
124. በግጭት ወይም በረብሻ ልጅዎን ተገድሎቦታል	0	1	99	
125. በግጭት ወይም በረብሻ ሌሳ የቤተሰብ አባል (ልጅ ወይም የትዳር ጓደኛ ያልሆነ) ተገድሎቦታል	0	1	99	
126. የትዳር ጓደኛዎ ታግተውብዎት ወይም የደረሱበት ጠፍቶ ያውቃል	0	1	99	
127. ልጅዎት ታግተውብዎት ወይም የደረሱበት ጠፍቶ ያውቃል	0	1	99	
128. ሌሳ የቤተሰብ አባል (ልጅ ወይም የትዳር ጓደኛ ያልሆነ) ታግተውብዎት ወይም የደረሱበት ጠፍቶ ያውቃል	0	1	99	
129. በጦርነት ወይም በፍንዳታ የተነሳ የቤተሰብዎ አባል ወይም ጓደኛዎ ሳይ የደረሰ ከባድ የመቁሰል አደጋ ነበር ወዪ	0	1	99	
130. አንድ ግለሰብ ጭንቅሳቱን ወይም ሌሳ አካሉን ሲደበደብ አይተው ያውቃሉ	0	1	99	
131. ሰው በግርፋት፡ኤሌክትሪክ ወዘተ በመሳሰሉት ስቃይ ሲደርስበት አይተው ያውቃሉ	0	1	99	
132. ከሳይ ከተዘረዘሩት አሰቃቂ ገጠመኞች በተጨማሪ በህይወት ዘመንዎ አጋጥሞዎት የሚያውቅ በጣም አስፈሪ ወይም ለህይወትዎ አስጊ ነገሮች ነበሩ ወዪ	0	1	99	

ካሱ --

STRESSFUL EVENTS
አስጨናቂ ገጠመኞችን በተመለከተ

ቃለ መጠይቅ ለሚደረጉ ሁሉ የሚከተሉት ጥያቄዎች ባለፉት 6 ወራት ወስጥ ስለ አጋጠሞት ሁኔታዎች ወይንም ድርጊቶች መጠየቅ እጀምራለው፡፡

	የለም	አዎ	ፈቃደኛ አይደሉም	
	0	1	99	
133. እርሶ ወይንም ከቤተሰቦ መሀል ሥራ ማጣት				
134. እርሶ ወይንም እቤቶ ውስጥ የሚኖሩ ቤተሰቦ በጠና መታመም	0	1	99	
135.በገንዘብ እጥረት ምክንያት የለት ተለት አስፈላጊ ነገሮች አለመሳካት	0	1	99	
136. እርሶ ወይንም እቤቶ ውስጥ የምኖሩ ቤተሰቦ የገንዘብ እዳ ወስጥ መግባት	0	1	99	
137. ከሰው ጋር ተሰዳድቦ መጣላት	0	1	99	
138. እረሶ ወይንም ቤቶ መዘረፍ	0	1	99	
139. ድብድብ ውስጥ መግባት ወይንም ሰው ሲደባደብ መመልከት	0	1	99	
140. ቤተሰቦ ውስጥ ሞት መከሠት	0	1	99	
141. ህግ ከመጣስ የትያያዙ ችግሮች	0	1	99	
142. አድስ ቤት መግባት	0	1	99	
143. ሌሎች ያጋጠሞት አስጨናቂ ገጠመኞች	0	1	99	

Specifiy ማብራሪያ---

ክፍል ፈ፡ የድህረ-አሰቃቂ ገጠመኝ ምልክቶች<br>ሰዎች መጥፎ ነገሮች በህይወታቸው ከገጠሟቸው ወይም አሰቃቂ ነገሮችን ከተመለከቱ የሚከተሉት የስነ-ልቦና ችግር ምልክቶች ሲታዩባቸው ይችላሉ፡፡ እያንዳንዱን ምልክት በጥንቃቄ ካደመጡ በኃላ እነዝህ ምልክቶች ባለፈው ሳምንት እርሶን ምን ያህል እንዳስጨነቁዎት ይጠቁሙ፡፡						
	በፍፁም	በመጠኑ	በከፍተኛ	በጣም ከፍተኛ	ፍቃደኛ አይደሉም	
144. ባለፈው ሳምንት የአስፈሪ ነገሮች/ድርጊቶች ትውስታ መደጋገም እርሶን ምን ያህል አስጨንቆት ነበር	1	2	3	4	99	
145. ባለፈው ሳምንት አሰቃቂወ ድርጊት ይደገማል ብሎ ማሰብ እርሶን ምን ያህል አስጨንቆት ነበር	1	2	3	4	99	
146. ባለፈው ሳምንት ተደጋጋሚ የሆነ ቅዠት መኖር እርሶን ምን ያህል አስጨንቆት ነበር	1	2	3	4	99	
147. ባለፈው ሳምንት ከሰዎች የመነጠል ስሜት መኖር እርሶን ምን ያህል አስጨንቆት ነበር	1	2	3	4	99	
148. ባለፈው ሳምንት ስሜት ማጣት እርሶን ምን ያህል አስጨንቆት ነበር	1	2	3	4	99	
149. ባለፈው ሳምንት ሆደ-ባሻ መሆን፡ በቀላሉ መከፋት ወይም መቆጣት እርሶን ምን ያህል አስጨንቆት ነበር	1	2	3	4	99	
150. ባለፈው ሳምንት ለነገሮች ትኩረት የመስጠት ችግር መኖር እርሶን ምን ያህል አስጨንቆት ነበር	1	2	3	4	99	
151. ባለፈው ሳምንት እንቅልፍ ማጣት ችግር መኖር እርሶን ምን ያህል አስጨንቆት ነበር	1	2	3	4	99	
152. ባለፈው ሳምንት ሰዎች ይከታተሉኛል ብሎ መስጋት እርሶን ምን ያህል አስጨንቆት ነበር	1	2	3	4	99	
153 ባለፈው ሳምንት. ግልፍተኛ ወይም ነጭናጫ በመሆኖ ምን ያህል አስጨንቆት ነበር	1	2	3	4	99	
154. ባለፈው ሳምንት አሰቃቂ ድርጊቶችን ሊያስታውሱ የሚችሉ ነገሮችን /ድርጊቶችን/ ለመከላከል ይሞክሩ ነበር	1	2	3	4	99	
155. ባለፈው ሳምንት አሰቃቂ ድርጊቶችን መይም ሁኔታዎች ለማስታወስ አለመቻል ምን ያህል አስጨንቆት ነበር	1	2	3	4	99	
156. ባለፈው ሳምንት በዕለታዊ ክንዋኔዎች ላይ ብዙም ፍላጐት አለማሳየ እርሶን ምን ያህል አስጨንቆት ነበር	1	2	3	4	99	
157. ባለፈው ሳምንት የወደፊት ተስፋ ማጣት ስሜት እርሶን ምን ያህል አስጨንቆት ነበር	1	2	3	4	99	
158. ባለፈው ሳምንት መጥፎ ገጠመኝን ሊያስታውሱ የሚችሉ ሃሣቦች ወይም ስሜቶችን ለማስወገድ ሞክረው ነበር	1	2	3	4	99	
159. ባለፈው ሳምንት መጥፎው/ዘግናኙ ድርጊት ሲታወስዎ የሚያሳዩት መንፈሳዊና አካላዊ ለውጥ መኖር እርሶን ምን ያህል አስጨንቆት ነበር	1	2	3	4	99	

	በፍፁም	በመጠኑ	በከፍተኛ	በጣም ከፍተኛ	ፍቃደኛ አይደሉም	
160. ባለፈው ሳምንት ቀድሞ ከነበረዎ ችሎታ የቀነሰ መምሰል እርሶን ምን ያህል አስጨንቆት ነበር	1	2	3	4	99	
161. ባለፈው ሳምንት ከአዳዲስ ሁኔታዎች ጋር ራስን በቀላሉ የማላመድ ችግር እርሶን ምን ያህል አስጨንቆት ነበር	1	2	3	4	99	
162. ባለፈው ሳምንት የድካም ስሜት መኖር እርሶን ምን ያህል አስጨንቆት ነበር	1	2	3	4	99	
163. ባለፈው ሳምንት የሰወነት ሕመም ስሜት መኖር እርሶን ምን ያህል አስጨንቆት ነበር	1	2	3	4	99	
164. ባለፈው ሳምንት አካላዊ መታወክ እርሶን ምን ያህል አስጨንቆት ነበር	1	2	3	4	99	
165. ባለፈው ሳምንት የማስታወስ ችሎታ መቀነስ እርሶን ምን ያህል አስጨንቆት ነበር	1	2	3	4	99	
166. ባለፈው ሳምንት ቀደም ብሎ የሠሩትን ሥራ ሌላ ሰው እስኪነግረዎት ድረስ አለማስታወስ ምን ያህል አስቸግሮት ነበር	1	2	3	4	99	
167. ባለፈው ሳምንት በነገሮች ላይ አትኩሮት ያለመስጠት ችግር እርሶን ምን ያህል አስጨንቆት ነበር	1	2	3	4	99	
168. ባለፈው ሳምንት ሁለት ስብዕና ኖርዎት፡ አንዱ እርሶዎ የሚያደርገትን ይመለከታል/ ይከታተላል/ እርሶን ምን ያህል አስጨንቆት ነበር	1	2	3	4	99	
169. ባለፈው ሳምንት የዕለታዊ ክንዋኔዎችን ማቀድ አለመቻል እርሶን ምን ያህል አስጨንቆት ነበር	1	2	3	4	99	
170. ባለፈው ሳምንት ለሆነው ነገር ሁሉ ራስዎን ይወቅሱ ነበር	1	2	3	4	99	
171. ባለፈው ሳምንት ከመጥፎው አደጋ በመትረፍዎ የሚሰማዎ የፀፀት ስሜት እርሶን ምን ያህል አስጨንቆት ነበር	1	2	3	4	99	
172. ባለፈው ሳምንት ተስፋ መቁረጥ ስሜት እርሶን ምን ያህል አስጨንቆት ነበር	1	2	3	4	99	
173. ባለፈው ሳምንት በገጠመዎ አሰቃቂ ድርጊት የተነሳ የመሸማቀቅ/ማፈር ስሜት እርሶን ምን ያህል አስጨንቆት ነበር	1	2	3	4	99	
174. ባለፈው ሳምንት የደረሰብዎትን ችግር ሰዎች አይረዱልኝም ብለው ማሰብ እርሶን ምን ያህል አስጨንቆት ነበር	1	2	3	4	99	
175. ሰዎች ይጠሉኛልን የሚል ስሜት መኖር እርሶን ምን ያህል አስጨንቆት ነበር	1	2	3	4	99	
176. ባለፈው ሳምንት የሚተማመኑበት ወይም ሃሣብዎን የሚያካፍሉት ሰው ያለዎ አይመስሉትም ነበር	1	2	3	4	99	
177. የሚያምኗቸው ሰዎች የከዱዎት ይመስልዎታል	1	2	3	4	99	
178. ባለፈው ሳምንት ባጋጠመዎ ነገር የተነሳ የበታችነት ስሜት መኖር እርሶን ምን ያህል አስጨንቆት ነበር	1	2	3	4	99	

	በፍፁም	በመጠኑ	በከፍተኛ	በጣም ከፍተኛ	ፍቃደኛ አይደሉም	
**179. በሌሎች ሰዎች ያልታመኑ ምን ያህል ይመስሎት ነበር**	**1**	**2**	**3**	**4**	**99**	
**180. ሌሎችን ለማገዝ ብርታት የማጣት ስሜት**	**1**	**2**	**3**	**4**	**99**	
**181. ባለፈው ሳምንት መጥፎ ድርጊት/ቶች ስምን ሲደርሱብዎ እንደቻሉ ብዙ ጊዜ ማስላሰል**	**1**	**2**	**3**	**4**	**99**	
**181. በዘግናኝ ድርጊቶች ውስጥ ያለፉ እርሶ ብቻ እንደሆኑ ማመን/ማሰብ**	**1**	**2**	**3**	**4**	**99**	
**182. ባለፈው ሳምንት የበቀለኝነት ስሜት መኖር እርሶን ምን ያህል ተፈታትኖት ነበር ,**	**1**	**2**	**3**	**4**	**99**	

**ክፍል ሰ፡ የሚከተሉትን ክትባቶች ልጆዎት ተከትቧል**	**ክትባት ካርድ**						
	**የለም**	**አዎ**		**የለም**	**አዎ**	**አላውቅም**	
**183. ልጆቾ ክትባት ተከትበዋል?**							
**የክትባቱ ከርድ አለዎት?**							
**184. ቢሲጂ፡ ፖሊዩ 0**	**0**	**1**		**0**	**1**	**88**	
**185. ዲፒቲ 1፡ ፖሊዩ 1**	**0**	**1**		**0**	**1**	**88**	
**186. ዲፒቲ 2፡ ፖሊዩ 2**	**0**	**1**		**0**	**1**	**88**	
**187. ዲፒቲ 3፡ ፖሊዩ 3**	**0**	**1**		**0**	**1**	**88**	
**188. የኩፍኝ ክትባት**	**0**	**1**		**0**	**1**	**88**	

189. እዚ የሚታየው የሥዕላዊ መግለጫ መሰላል በእርሶ ቀበሌ ውስጥ ነዋሪዎችን እንደሚመስል ያስቡና.
እሱን በተመለከተ ጥያቄ እጠይቆጣለው፡፡

ሥዕሉ መሰላል ጫፍ የሚያመለክተው በዚ ቀበሌ ውስጥ ያሉ ሀብትም - ገንዘብና ቤት ያላቸውን ያመለከታል. መሰላሉ ሥር ደሞ በዚ ቀበሌ ውስጥ ያሉ ድሃ ወይንም ገንዘብና ቤት የሌላቸውን ሰዎችን ይመለከታል፡፡ ወደ መሰላሉ ጫፍ መቅረብ ወደ ገንዘብ ወይንም ቤት ያላቸው ሰዎችን መቅረብ ያመለክታል፡፡ ወደ መሰላሉ ሥር መቅረብ ወደ ደሞ ድሃነት ወይንም ገንዘብና ቤት የሌላቸውን ሰዎችን መቅረብን ያመለክታል፡፡

ባሁኑ ጊዜ የሕወቶ ደረጃ በቀበሌዎት ካሉ ሰዎች ጋር ሲነጻጸር ብለው የሚያስቡትን የሥዕሉ መሰላል ደረጃ ላይ በ X ያስምሩበት ፡፡ ቁጥሩን በወረቀቱ ያስፍሩበት

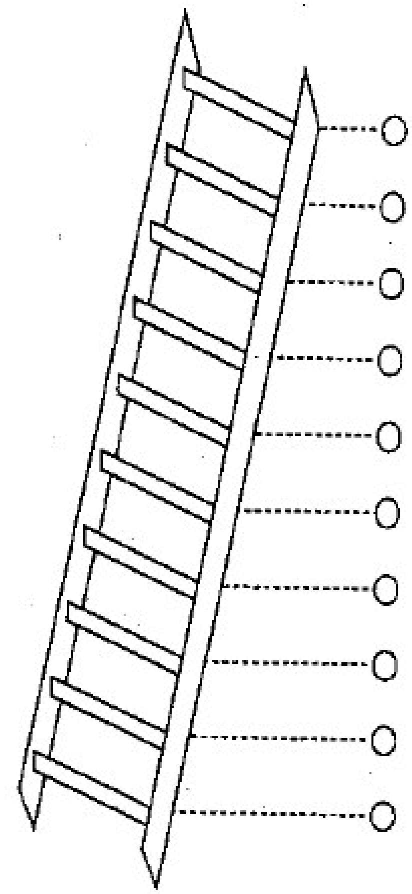

የተለያዩ የምርጫ ሙከራዎችን በተመለከተ:: **DISCRETE CHOICE EXPERIMENT**

አሁን ስምንት ካርዶች አሳዮታለሁ:: እያንዳንዱ ካርድ ስለ ሁለት ጤና ጣቢያ የሚገልጽ ነው:: አሁን በጠና ቢታመሙ ብለው ያስቡና የትኛው የጤና ጣብያ ውስጥ ቢታከሙ እንደሚመርጡ ይንገሩኝ:: ሁለቱንም ጤና ጣብያ አለመጠቀም ምርጫ አሎት:: በነዚህ ጥያቄዎች ሳይ ትክክል ወይንም የተሳሳተ መልስ የለም:: ከነዘኚ ጥያቄዎች ማወቅ የምንፈልገው ምን ያህል ጤና ጣብያ ውስጥ መታከም አስፈሳጊ ነው ብለው የሚያምኑበትን ነገሮች ስማየት ነው::

ሁለቱንም ጤና ጣብያ አለመጠቀም ምርጫ አሎት:: በዚህ ጥያቄ ሳይ ትክክል ወይንም የተሳሳተ መልስ የለም:: ከነዚ ጥያቄዎች ማወቅ የምንፈልገው ምን ያህል ጤና ጣብያ ውስጥ አስፈሳጊ ብለው የሚያምኑበትን ነገሮች ስማይት ነው::

	ጤና ጣብያ (ሀ)	ጤና ጣብያ (ለ)	ወደ ሁሉቱመ አለሄድም	ፍቃደኛ አይደለሁም
ካርድ1	1	2	3	99
ካርድ2	1	2	3	99
ካርድ3	1	2	3	99
ካርድ4	1	2	3	99
ካርድ5	1	2	3	99
ካርድ6	1	2	3	99
ካርድ7	1	2	3	99
ካርድ8	1	2	3	99

ለኢንተርቭው ለምያደርገው ሰው:: የተጠቀሙትን የካርድ አይነቱን ቁጥር ያስምሩበት 1 2 3 4 5
INTERVIEWER: PLEASE CIRCLE THE VERSION NUMBER USED: 1. 2. 3. 4. 5

መጠነሰብ				
ህፃን	ክብደት		ቁመት/ርዝመት/	
እናት	ክብደት		ቁመት	

አመሰግናለሁ!!
