# Supplementary material for: Household food insecurity and symptoms of neurologic disorder in Ethiopia: An observational analysis
Source: BMC Public Health. 2010 Dec 31;10:802. doi: 10.1186/1471-2458-10-802 (PMC3027184; doi:10.1186/1471-2458-10-802)
Supplement: Additional file 3 — Survey questionnaire for men in English. The survey questionnaire used to obtain data about men in the analysis translated in English. [file 1471-2458-10-802-S3.DOC]

**Research project on “Child health, development and adult mental health”**

**Confidential: only used for research purposes**

| Name of interviewer | | Kebele name --------------- | | | Household ID ----------------- | |
| --- | --- | --- | --- | --- | --- | --- |
| Interviewer signature : | |
| Name of supervisors | | House number ----------- | | | Father ID ---------------- | |
| Supervisors signature : | |  | | |
| Outcome of first visit | 1.Completed 2. Not around home 3. Interrupted 4. Refused | |  | Date of appointment for other time visit | | |
| Outcome of second visit | 1.Completed 2. Not around home 3. Interrupted 4. Refused | |  | Date of appointment for other time visit | | |
| Outcome of third Visit | 1.Completed 2. Not around home 3. Interrupted 4. Refused | |  | Date of appointment for other time visit | | |
| Date of interview date/month/ year | -------- /--------/----------- | | Time of start of interview | | |  |

| **ANXIETY AND DEPRESSION**  We would like to ask you questions about your past history and present symptoms. This information will be used to help us provide you with better medical care. However, you may find some questions upsetting. If so, please feel free not to answer. The answers to the questions will be kept confidential. Listed below are symptoms or problems that people sometimes have. Please read each one carefully and describe how much the symptoms bothered you or distressed you in the last week, including today. | | | | | | |
| --- | --- | --- | --- | --- | --- | --- |
|  | **not at all** | **a little bit** | **very much** | **extremely** | **refused** |  |
| 1. In the past week how bothered have you been by feeling suddenly scared for no reason? | 0 | 1 | 2 | 3 | 99 |  |
| 1. In the past week how bothered have you been by feeling fearful? | 0 | 1 | 2 | 3 | 99 |  |
| 1. In the past week how bothered have you been by faintness, dizziness or weakness? | 0 | 1 | 2 | 3 | 99 |  |
| 1. In the past week how bothered have you been by nervousness or shakiness inside? | 0 | 1 | 2 | 3 | 99 |  |
| 1. In the past week how bothered have you been by your heart pounding or racing? | 0 | 1 | 2 | 3 | 99 |  |
| 1. In the past week how bothered have you been by trembling? | 0 | 1 | 2 | 3 | 99 |  |
| 1. In the past week how bothered have you been by feeling tense or keyed up? | 0 | 1 | 2 | 3 | 99 |  |
| 1. In the past week how bothered have you been by headaches? | 0 | 1 | 2 | 3 | 99 |  |
| 1. In the past week how bothered were you by a spell of terror or panic? | 0 | 1 | 2 | 3 | 99 |  |
| 1. In the past week how bothered have you been by feeling like you can’t sit still or restlessness? | 0 | 1 | 2 | 3 | 99 |  |
| 1. In the past week how bothered were you by feeling low in energy, slowed down? | 0 | 1 | 2 | 3 | 99 |  |
| 1. In the past week how bothered were you by blaming yourself for things? | 0 | 1 | 2 | 3 | 99 |  |
| 1. In the past week how bothered were you by crying easily? | 0 | 1 | 2 | 3 | 99 |  |
| 1. In the past week how bothered were you by a loss of sexual interest or pleasure? | 0 | 1 | 2 | 3 | 99 |  |
| 1. In the past week how bothered were you by a poor appetite? | 0 | 1 | 2 | 3 | 99 |  |
| 1. In the past week how bothered were you by difficulty falling asleep or staying asleep? | 0 | 1 | 2 | 3 | 99 |  |
| 1. In the past week how bothered were you by feeling hopeless about the future? | 0 | 1 | 2 | 3 | 99 |  |
| 1. In the past week, have you been feeling blue? | 0 | 1 | 2 | 3 | 99 |  |
| 1. In the past week how bothered were you by feeling lonely? | 0 | 1 | 2 | 3 | 99 |  |
| 1. In the past week have you thought of ending your life? | 0 | 1 | 2 | 3 | 99 |  |
| 1. In the past week how bothered were you by feelings of being trapped or caught? | 0 | 1 | 2 | 3 | 99 |  |
| 1. In the past week how bothered were you by too much about things? | 0 | 1 | 2 | 3 | 99 |  |
| 1. In the past week, how bothered were you by feeling no interest in things? | 0 | 1 | 2 | 3 | 99 |  |
| 1. In the past week how bothered were you by feeling everything is an effort? | 0 | 1 | 2 | 3 | 99 |  |
| 1. In the past week how bothered were you by feelings of worthlessness? | 0 | 1 | 2 | 3 | 99 |  |

| **SOCIOECONOMIC STATUS** | | | | | | | | |
| --- | --- | --- | --- | --- | --- | --- | --- | --- |
|  | | | | **No** | **Yes** | | **Refused** |  |
| 1. In the last three months, did you ever worry that your household would not have enough food? | | | | 0 | 1 | | 99 |  |
| 1. In the last three months, did you ever reduced of balanced diet to your child because of lack of food or money to buy | | | | 0 | 1 | | 99 |  |
| 1. In the last three months, did you ever reduced of balanced diet to your child because of lack of food or money to buy | | | | 0 | 1 | | 99 |  |
| 1. In the last three months, did you ever reduce the number of meals for in your child a day because of shortage of food or money | | | | 0 | 1 | | 99 |  |
| 1. In the last three months, did you ever reduce the number of meals of eaten child a day because of shortage of food or money | | | | 0 | 1 | | 99 |  |
| 1. In the last three months, did you ever spend the whole day without eating because of shortages of food or money | | | | 0 | 1 | | 99 |  |
| 1. In the last three months, did you ever ask for food or money to buy food? | | | | 0 | 1 | | 99 |  |
| 1. Do you own your house? | | | | 0 | 1 | | 99 |  |
| 1. Do you have land to plant or farm? | | | | 0 | 1 | | 99 |  |
| 1. Does household have a bicycle? | | | | 0 | 1 | | 99 |  |
| 1. Do you have radio? | | | | 0 | 1 | | 99 |  |
| 1. Do you have electricity? | | | | 0 | 1 | | 99 |  |
| 73a. Do you have ox? | | | | 0 | 1 | | 99 |  |
| 73b. Do you have cow? | | | | 0 | 1 | | 99 |  |
| 73c. Do you hen? | | | | 0 | 1 | | 99 |  |
| 73d. Do you have Sheep or goat | | | | 0 | 1 | | 99 |  |
| 73e. Do you have telephone or Mobile? | | | | 0 | 1 | | 99 |  |
| 73f. Do you have Television? | | | | 0 | 1 | | 99 |  |
| 73g. Do you have Donkey? | | | | 0 | 1 | | 99 |  |
| 73h. Do you have horse or mule? | | | | 0 | 1 | | 99 |  |
| 73i. Do you have economic trees such as Mango, Papaya, Coffee | | | | 0 | 1 | | 99 |  |
| 73J. Do you have tape or DVD/VHS player | | | | 0 | 1 | | 99 |  |
| 1. Household floor material | | 1. earthen | 2. wood | | | 3.Cement/file | |  |
| 74a. Household roof material | | 1. corrigated iron sheet | 2. thatched | | | 3. concert | |  |
| 74b. Household wall material | | 1. Mud | 2. Cement/file | | | 3. wood | |  |
| 74c source of drinking water | 1. Pipe | 2. Protected spring or well | 3. Unprotected spring or well | | | 4. River | |  |
| 74D. toilet facility | | 1. Private | 2. Shared | | | 3. Use open field | |  |

| **TRAUMA EVENTS**  Please indicate whether you have experienced any of the following events (check Yes or No) | | | | |
| --- | --- | --- | --- | --- |
|  | No | Yes | Refused |  |
| 1. Lack of shelter | 0 | 1 | 99 |  |
| 1. Lack of food or water | 0 | 1 | 99 |  |
| 1. Ill health without access to medical care | 0 | 1 | 99 |  |
| 1. Confiscation or destruction of personal property | 0 | 1 | 99 |  |
| 1. Combat situation (e.g. shelling or grenades) | 0 | 1 | 99 |  |
| 1. Forced evacuation under dangerous conditions | 0 | 1 | 99 |  |
| 1. Beating to the body | 0 | 1 | 99 |  |
|  | 0 | 1 | 99 |  |
|  | 0 | 1 | 99 |  |
|  | 0 | 1 | 99 |  |
| 1. Knifing or axing | 0 | 1 | 99 |  |
| 1. Torture, i.e., while in captivity you received deliberate and systematic infliction of physical or mental suffering | 0 | 1 | 99 |  |
| 1. Serious physical injury from combat situation or landmine | 0 | 1 | 99 |  |
| 1. Imprisonment | 0 | 1 | 99 |  |
| 1. Forced labor (like animal or slave) | 0 | 1 | 99 |  |
| 1. Extortion or robbery | 0 | 1 | 99 |  |
| 1. Brainwashing | 0 | 1 | 99 |  |
| 1. Forced to hide | 0 | 1 | 99 |  |
| 1. Kidnapped | 0 | 1 | 99 |  |
| 1. Other forced separation from family members | 0 | 1 | 99 |  |
| 1. Forced to find and bury bodies | 0 | 1 | 99 |  |
| 1. Enforced isolation from others | 0 | 1 | 99 |  |
| 1. Someone was forced to betray you and placed you at risk of death or injury | 0 | 1 | 99 |  |
| 1. Prevented from burying someone | 0 | 1 | 99 |  |
| 1. Forced to desecrate or destroy the bodies or graves of deceased persons | 0 | 1 | 99 |  |
| 1. Forced to physically harm family member, or friend | 0 | 1 | 99 |  |
| 1. Forced to physically harm someone who is not a family member, or friend | 0 | 1 | 99 |  |
| 1. Forced to destroy someone else's property or possessions | 0 | 1 | 99 |  |
| 1. Forced to betray family member, or friend placing them at risk of death or injury | 0 | 1 | 99 |  |
| 1. Forced to betray someone who is not family or friend placing them at risk of death or injury | 0 | 1 | 99 |  |
| 1. Murder, or death due to violence, of spouse | 0 | 1 | 99 |  |
| 1. Murder, or death due to violence, of child | 0 | 1 | 99 |  |
| 1. Murder, or death due to violence, of other family member or friend | 0 | 1 | 99 |  |
| 1. Disappearance or kidnapping of spouse | 0 | 1 | 99 |  |
| 1. Disappearance or kidnapping of child | 0 | 1 | 99 |  |
| 1. Disappearance or kidnapping of other family member or friend | 0 | 1 | 99 |  |
| 1. Serious physical injury of family member or friend due to combat situation or landmine | 0 | 1 | 99 |  |
| 1. Witness beatings to head or body | 0 | 1 | 99 |  |
| 1. Witness torture | 0 | 1 | 99 |  |
| 1. Any other situation that was very frightening or felt your life was in danger. | 0 | 1 | 99 |  |
| Specify --------------------------------------------------------------------------------------------------------------------------------------------------------------------------------------------------------------------------------------------------------------------------------------------------------------------------- | | | | |

| **Pots Traumatic Stress Disorder**  The following are symptoms that people have after experiencing hurtful or terrifying events in their lives. Please read each one carefully and decide how much the symptoms bothered you **IN THE PAST WEEK** | | | | | | |
| --- | --- | --- | --- | --- | --- | --- |
|  | **Not at all** | **Little** | **Quite a bit** | **Extremely** | **Refused** |  |
| 1. Recurrent thoughts or memories of the most hurtful or terrifying events. | 0 | 1 | 2 | 3 | 99 |  |
| 1. Feeling as though the event is happening again. | 0 | 1 | 2 | 3 | 99 |  |
| 1. Recurrent nightmares. | 0 | 1 | 2 | 3 | 99 |  |
| 1. Feeling detached or withdrawn from people. | 0 | 1 | 2 | 3 | 99 |  |
| 1. Unable to feel emotions. | 0 | 1 | 2 | 3 | 99 |  |
| 1. Feeling jumpy, easily started. | 0 | 1 | 2 | 3 | 99 |  |
| 1. Difficulty concentrating. | 0 | 1 | 2 | 3 | 99 |  |
| 1. Trouble sleeping. | 0 | 1 | 2 | 3 | 99 |  |
| 1. Feeling on guard. | 0 | 1 | 2 | 3 | 99 |  |
| 1. Feeling irritable or having outburst of anger | 0 | 1 | 2 | 3 | 99 |  |
| 1. Avoiding activities that remind you of the traumatic or hurtful event. | 0 | 1 | 2 | 3 | 99 |  |
| 1. Inability to remember parts of the most traumatic or hurtful events | 0 | 1 | 2 | 3 | 99 |  |
| 1. Less interest in daily activities | 0 | 1 | 2 | 3 | 99 |  |
| 1. Feeling as if you don't have a future | 0 | 1 | 2 | 3 | 99 |  |
| 1. Avoiding thoughts or feelings associated with the traumatic or hurtful events | 0 | 1 | 2 | 3 | 99 |  |
| 1. Sudden emotional or physical reaction when reminded of the most hurtful or traumatic events | 0 | 1 | 2 | 3 | 99 |  |
| 1. Feeling that you have less skills than you had before | 0 | 1 | 2 | 3 | 99 |  |
| 1. Having difficulty dealing with new situations | 0 | 1 | 2 | 3 | 99 |  |
| 1. Feeling exhausted | 0 | 1 | 2 | 3 | 99 |  |
| 1. Bodily pain | 0 | 1 | 2 | 3 | 99 |  |
| 1. Troubled by physical problem(s) | 0 | 1 | 2 | 3 | 99 |  |
| 1. Poor memory | 0 | 1 | 2 | 3 | 99 |  |
| 1. Finding out or being told by other people that you have done something that you cannot remember | 0 | 1 | 2 | 3 | 99 |  |
| 1. Difficulty paying attention | 0 | 1 | 2 | 3 | 99 |  |
| 1. Feeling as if you are split into two people and one of you is watching what the other is doing | 0 | 1 | 2 | 3 | 99 |  |
| 1. qFeeling unable to make daily plans | 0 | 1 | 2 | 3 | 99 |  |
| 1. Blaming yourself for things that have happened | 0 | 1 | 2 | 3 | 99 |  |
| 1. Feeling guilty for having survived. | 0 | 1 | 2 | 3 | 99 |  |
| 1. Hopelessness | 0 | 1 | 2 | 3 | 99 |  |
| 1. Feeling ashamed of the hurtful or traumatic events that have happened to you | 0 | 1 | 2 | 3 | 99 |  |
| 1. Feeling that people do not understand what happened to you | 0 | 1 | 2 | 3 | 99 |  |
| 1. Feeling others are hostile to you | 0 | 1 | 2 | 3 | 99 |  |
| 1. Feeling that you have no one to rely upon | 0 | 1 | 2 | 3 | 99 |  |
| 1. Feeling that someone you trusted betrayed you | 0 | 1 | 2 | 3 | 99 |  |
| 1. Feeling humiliated by your experience | 0 | 1 | 2 | 3 | 99 |  |
| 1. Feeling no trust in others | 0 | 1 | 2 | 3 | 99 |  |
| 1. Feeling powerless to help others | 0 | 1 | 2 | 3 | 99 |  |
| 1. Spending time thinking why these events happened to you | 0 | 1 | 2 | 3 | 99 |  |
| 1. Feeling that you are the only one that suffered these events. | 0 | 1 | 2 | 3 | 99 |  |
| 1. Feeling a need for revenge. | 0 | 1 | 2 | 3 | 99 |  |

| **ADULT NEUROLOGICAL CONDITIONS** | | | | | |
| --- | --- | --- | --- | --- | --- |
|  | **No** | **Yes** | **Don't Know** | **Refused** |  |
| 1. Have you ever suffered a loss of consciousness from being struck in the head or in an accident | 0 | 1 | 88 | 99 |  |
| 1. Do you have numbness or loss of sensation in your hands | 0 | 1 | 88 | 99 |  |
| 1. Do you have numbness or loss of sensation in your feet | 0 | 1 | 88 | 99 |  |
| 1. Have you ever had a seizure (an episode where you lost consciousness and had uncontrolled shaking) | 0 | 1 | 88 | 99 |  |
| 1. Do you occasionally have weakness in an arm that eventually returns to normal | 0 | 1 | 88 | 99 |  |
| 1. Do you occasionally have weakness in a leg that eventually returns to normal | 0 | 1 | 88 | 99 |  |
| 1. Do you have permanent weakness in an arm or hand | 0 | 1 | 88 | 99 |  |
| 1. Do you have permanent weakness in your face | 0 | 1 | 88 | 99 |  |
| 1. Do you have permanent weakness in a leg or foot | 0 | 1 | 88 | 99 |  |
| 1. Do you have blurry vision | 0 | 1 | 88 | 99 |  |
| 1. Do you ever have temporary loss of vision | 0 | 1 | 88 | 99 |  |
| 1. Do you ever have shaking in an arm or leg even when you keep them still | 0 | 1 | 88 | 99 |  |
| 1. Do you ever have bothersome shaking in an arm or hands when you are reaching for objects | 0 | 1 | 88 | 99 |  |
| 1. Have you ever had difficulty speaking that eventually returned to normal | 0 | 1 | 88 | 99 |  |
| 1. Do you have permanent difficulties speaking | 0 | 1 | 88 | 99 |  |
| 1. Do you walk with a limp | 0 | 1 | 88 | 99 |  |
| 1. Do you have back pain on a regular basis | 0 | 1 | 88 | 99 |  |
| 1. Do you have neck pain on a regular basis | 0 | 1 | 88 | 99 |  |
| 1. Do you have pain that radiates across the face | 0 | 1 | 88 | 99 |  |
| 1. Do you have uncontrolled jerking/twitching of your face | 0 | 1 | 88 | 99 |  |
| 1. Do you ever have pain in your hands that wakes you from sleep | 0 | 1 | 88 | 99 |  |
| 1. I should this be DO YOU frequently loose control of my bowel or bladder | 0 | 1 | 88 | 99 |  |

| 199. **Think of this ladder as representing where people stand in your kebele**  At the top of the ladder are people who very very rich- those who have most money and household assets. At the bottom are those who are very very poor- those who have least money and household assets. The higher up you are in this ladder, the closer you are to the people at the top. The lower down you are, the closer you are to the people the very bottom.  Please put an X mark on the rung you think and you stand at this time in your life, relative to other people in your kebele or community. Write the number on in the blank space |
| --- |


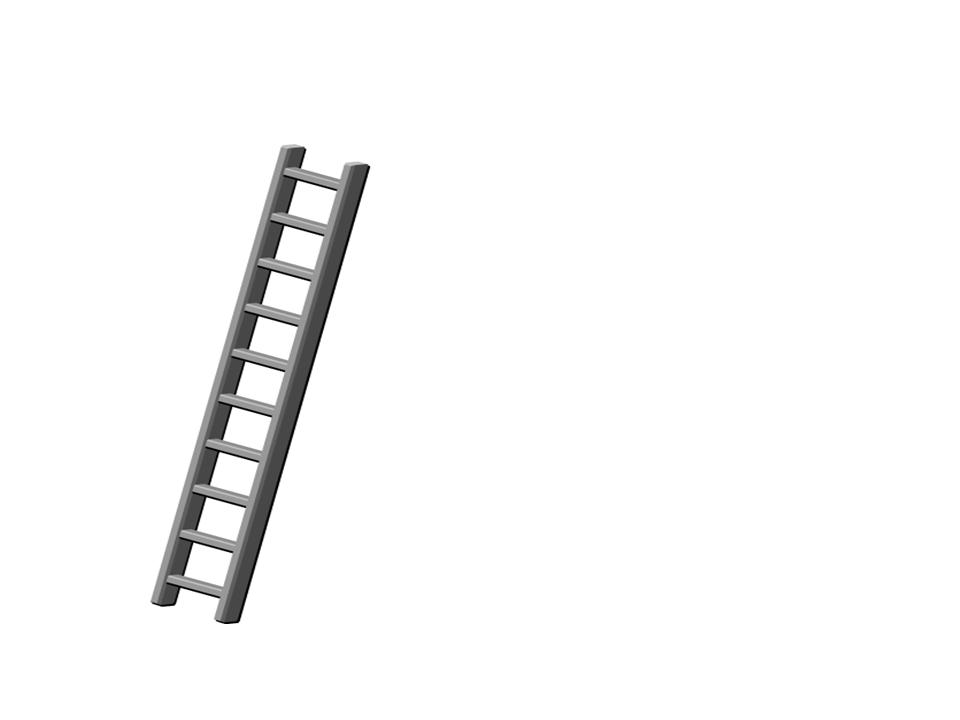


10th rank– Very very reach

1^st^— very very poor

| **ANTHROPOMETRY** | | | | |
| --- | --- | --- | --- | --- |
| 200. Father | weight |  | Height |  |

Thank you!!

Interview indeed (HH:MM) ---------------------------------------
