# Supplementary material for: Household food insecurity and symptoms of neurologic disorder in Ethiopia: An observational analysis
Source: BMC Public Health. 2010 Dec 31;10:802. doi: 10.1186/1471-2458-10-802 (PMC3027184; doi:10.1186/1471-2458-10-802)
Supplement: Additional file 4 — Survey questionnaire for men in Amharic. The survey questionnaire used to obtain data about men in the analysis in Amharic. [file 1471-2458-10-802-S4.PDF]

ሚስጥራዊነቱ የተጠበቀና ለጥናት ብቻ የሚውል መረጃ

የመረጃ ሰብሳቢው ስም		የቀበሌው ስም -------------------		የቤተሰብ መለያ ቁጥር:-----------
የተቆጣጣሪው ስም		የቤት ቁጥር ---------------------		የእናት መለያ ቁጥር-------------
የተቆጣጣሪው ፊርማ፡				የህፃኑ/ኗ መለያ ቁጥር------------
የመጀመሪያው ጉብኝት ውጤት	1 ተጠናቋል 2 በቤት የሉም<br>3 ፍቃደኛ አልሆኑም 4 ተቋርጧል			ተለዋጭ የቃለመጠይቅ ቀጠሮ የተያዘበት ቀን፡
የሁለተኛው ጉብኝት ውጤት	1 ተጠናቋል 2 በቤት የሉም<br>3 ፍቃደኛ አልሆኑም 4 ተቋርጧል			ተለዋጭ የቃለመጠይቅ ቀጠሮ የተያዘበት ቀን፡
የሦስተኛው ጉብኝት ውጤት	1 ተጠናቋል 2 በቤት የሉም<br>3 ፍቃደኛ አልሆኑም 4 ተቋርጧል			ተለዋጭ የቃለመጠይቅ ቀጠሮ የተያዘበት ቀን፡
ቃለ መጠይቁ የተካሄደበት ቀን/ወር/ ዓም	-------- /--------/-----------	መጠይቁ የተጀመረበት ሰዓት፡		

Age and schooling	
1.እድሜዎ ስንት ነው?	------------------- አመታት
2. ትምህርት ለስንት አመታት ያሕል ተምረው ያውቃሉ?	------------------- አመታት

These questions are about the health care facilities nearest you<br>ከዚህ በታች ያሉት ጥያቄዎች ባካባበዎ ስላሉ የህክምና ጣብዎች የሚመለከቱ ናቸው		
3. ለእርሶ የሚቀርቦት የጤና ጣቢያ በየት ይገኛል?	______________ሰፍራ/ቦታ	
4. ከ 0 እስከ 99 ባለው መለኪያ፣ ጤና ጣቢያው ምን ያህል የአገልግሎት ጥራት አለው ብለው ይገምታሉ?	0 እጅግ በጣመ ጥሩ<br>1 በጣም ጥሩ<br>2 ጥሩ<br>3 መደበኛ<br>4 የደከመ<br>99 ፍቃደኛ አይደሉም	
5. ለእርሶ የሚቀርቦት የጤና ማዕከል በየት ይገኛል?	______________ ሰፍራ/ቦታ	
6. ከ 0 እስከ 99 ባለው መለኪያ፣ ጤና ማዕከሉ ምን ያህል የአገልግሎት ጥራት አለው ብለው ይገምታሉ?	0 እጅግ በጣመ ጥሩ<br>1 በጣም ጥሩ<br>2 ጥሩ<br>3 መደበኛ<br>4 የደከመ<br>99 ፍቃደኛ አይደሉም	
7. ለእርሶ የሚቀርቦት ሆስፒታል በየት ይገኛል?	______________ ሰፍራ/ቦታ	
8. ከ 0 እስከ 99 ባለው መለኪያ፣ሆስፒታሉ ምን ያህል የአገልግሎት ጥራት አለው ብለው ይገምታሉ?	0 እጅግ በጣመ ጥሩ<br>1 በጣም ጥሩ<br>2 ጥሩ<br>3 መደበኛ<br>4 የደከመ<br>99 ፍቃደኛ አይደሉም	

9. ከ 0 እስከ 99 ባለው መለኪያ፣ ዛሬ ጤናዎትን በምን ያክል መጠን ያዩታል?	0 እጅግ በጣመ ጥሩ<br>1 በጣም ጥሩ<br>2 ጥሩ<br>3 መደበኛ<br>4 የደከመ<br>99 ፍቃደኛ አይደሉም	
10. ባለፉት አምስት አመታት ውስጥ በጠና የታመሙበት ጊዜ ነበር?	1 yes አዎ<br>2 no የለም<br>99 አላውቅም/ፍቃደኛ አይደለሁም	
11. በቅርብ የታመሙበትን ጊዜ ያስቡና የበሽታዎ ወይንም ቁስሉ የተሰማዎትን ህመም ይንገሩኝ?	1 ከሳንባ ጋር የተያያዘ(ሳል, ትንፋሽ ማጠር)<br>2 [illegible] በሽታ (ሆድ ቁርተት)<br>3 ትኩሳት ወይንም የመጣል በሽታ አይነት<br>4 መቁሰል (መድማት, የአጥንት መሰበር)<br>5 ሌላ (ዝርዝር)<br>99 አላውቅም/ፍቃደኛ አይደሉም	
12. ለዚ ህመምዎ ወደ ሀኪም ቤት ሄደው ያውቃሉ?	1 አዎ<br>2 የለም<br>99 አላውቅም/ፍቃደኛ አይደሉም	
መልሱ የለም ከሆነ ወደ ጥያቄ ቁጥር 15 ያምሩ፡ መልሱ አዎ ከሆነ ወደ ጥያቄ ቁጥር 13 ያምሩ		
13. የትኛው ሀኪም ቤት ነው የሄዱት?<br>ስምና ቦታ	1 ስም (ቦታ)________________________________	
14. ምን አይነት ሀኪም ቤት ነበር?	1 የመንግስት ጤና ጣቢያ<br>2 የመንግስት ጤና ማዕከል<br>3 የመንግስት ሆስፒታል<br>4 የሚሽን ጤና ጣብያ<br>5 የሚሽን ጤና ማዕከል<br>6 የሚሽን ሆስፒታል<br>7 የግል ጤና ጣብያ<br>8 የግል ጤና ማዕከል<br>9 የግል ሆስፒታል<br>10 ሌላ(ዝርዝር)<br>99 አላቅም	

ክፍል ሐ፡ ጭንቀትና ድብርትን በተመለከተ

ስላለፈው ህይወትዎ እና በአሁን ሰዓት ስላሉት አንዳንድ የጭንቀትና ድብርት ምልክቶች ልንጠይቅዎ እንፈልጋለን፡፡ እርስዎ በግልፅነት የሚሰጡን መረጃ ተገቢ የሆነ የህክምና አገዛ እንዲደረግልዎት ይረዳናል፡፡ ለጥያቄዎቹ የሚሰጡን መልስ ሚስጥራዊነቱ የተጠበቀ ነው፡፡ ቀጥሎ የተዘረዘሩት የጭንቀት ወይም የድብርት ምልክቶች በብዙ ሰዎች ዘንድ አንዳንዴ የተለመዱ ናቸው፡፡ እያንዳንዱን ጥያቄ በጥንቃቄ ካዳመጡ በኃላ <u>ሳለፈው ሳምንት /ዛሬን ጨምሮ/</u> ምን ያህል እርሶን እንዳወከዎት ይግለፁ፡

	በፍፁም	በመጠኑ	በከፍተኛ	በጣም ከፍተኛ	ፍቃደኛ አይደሉም	
15. ባለፈው ሳምንት ያለምንም ምክንያት ድንገት ውስጥዎን ፍርሃት ፍርሃት ይለው ነበር	0	1	2	3	99	
16. ባለፈው ሳምንት በፍርሃት ስሜት የተነሳ ምን ያህል አስበዎል	0	1	2	3	99	

	በፍፁም	በመጠኑ	በከፍተኛ	በጣም ከፍተኛ	ፍቃደኛ አይደሉም	
17. ባለፈው ሳምንት የድካም፡ የመዝለፍለፍ ወይም ራስዎን የመክበድ ስሜትዎ ምን ያህል አሳስቦታል	0	1	2	3	99	
18. ባለፈው ሳምንት የመረበሽ ወይም የመፍራት ስሜትዎስ ምን ያህል አሳስብዎት ነበር	0	1	2	3	99	
19. ባለፈው ሳምንት ልብዎ በሀይል ወይም ቶሎ ቶሎ መምታት ምን ያህል አሳስብዎት ነበር	0	1	2	3	99	
20. ባለፈው ሳምንት ሰውነትዎ በፍርሃት ይንቀጠቀጥብዎ ነበር	0	1	2	3	99	
21. ባለፈው ሳምንት የሰውነትዎ መጨናነቅ ስሜትዎ ምን ያህል አሳስብዎት ነበር	0	1	2	3	99	
22. ባለፈው ሳምንት የራስ ምታት መኖር ምን ያህል አሳስብዎት ነበር	0	1	2	3	99	
23. ባለፈው ሳምንት በአጋጣም መሸበር ወይም ከፍተኛ ፍርሓት ስሜትዎስ ምን ያህል አሳስብዎት ነበር	0	1	2	3	99	
24. ባለፈው ሳምንት ተረጋግቶ ያስመቀመጥ ወይም ዕረፍት የማጣት ስሜትዎስ ምን ያህል አሳስብዎት ነበር	0	1	2	3	99	
25. ባለፈው ሳምንት ብርታት/አቅም የማጣት ስሜትዎስ ምን ያህል አሳስብዎት ነበር	0	1	2	3	99	
26. ባለፈው ሳምንት በሆነ ባልሆነው ራስዎን ይወቅሱ ነበር	0	1	2	3	99	
27. ባለፈው ሳምንት በቀላሉ አልቅስ/ሽ አልቅስ/ሽ የሚል ስሜት ምን ያህል አሳስብዎት ነበር	0	1	2	3	99	
28. ባለፈው ሳምንት የፍትዎት ስሜት ማጣት ምን ያህል አሳስብዎት ነበር	0	1	2	3	99	
29. ባለፈው ሳምንት የምግብ ፍላጐት ማጣትስ ምን ያህል አሳስብዎት ነበር	0	1	2	3	99	
30. ባለፈው ሳምንት የእንቅልፍ ማጣት ችግር ምን ያህል አሳስብዎት ነበር	0	1	2	3	99	
31. ባለፈው ሳምንት የተስፋ መቁረጥ ስሜትዎስ ምን ያህል አሳስብዎት ነበር	0	1	2	3	99	
32. ባለፈው ሳምንት አዝነው ወይም ተክዘው ነበር	0	1	2	3	99	
33. ባለፈው ሳምንት የብቸኝነት ስሜትስ ምን ያህል አሳስብዎት ነበር	0	1	2	3	99	
34. ባለፈው ሳምንት ህይወትዎን የማጥፋት ሀሣብ ይመጣብዎ ነበር	0	1	2	3	99	
35. ባለፈው ሳምንት በስራም ይሁን በሌላ ጉዳይ የመጠመድ ስሜት ምን ያህል አሳስብዎት ነበር	0	1	2	3	99	
36. ባለፈው ሳምንት ስነገሮች አብዝተው ይጨነቁ ነበር	0	1	2	3	99	
37. ባለፈው ሳምንት ስነገሮች ሁሉ ስምንም ዓይነት ፍላጐት ማጣት ስሜት ምን ያህል አሳስብዎት ነበር	0	1	2	3	99	

	በፍፁም	በመጠኑ	በከፍተኛ	በጣም ከፍተኛ	ፍቃደኛ አይደሉም	
38. ማንኛው ነገር የሚገኘው ወይም የሚሳካው በድካም ነው እያሉ ምን ያህል ተጨንቋዋል	0	1	2	3	99	
39. ባለፈው ሳምንት የዋጋ-ቢስነት ስሜትስ ምን ያህል አሳስብዎት ነበር.	0	1	2	3	99	

ክፍል መ፡ ማህበራዊና ኢኮኖሚያዊ ሁኔታን በተመለከተ			
	የለም	አዎ	
40. ባለፉት ሦስት ወራት ውስጥ ቤተዎ ውስጥ በቂ ምግብ አይኖርም ብለው ተጨንቀው ያውቀሉ ወይ?	0	1	
41. ባለፉት ሦስት ወራት ውስጥ እርሶ ወይንም ቤተሰብዎ ተጨማሪ የምግብ መግዣ ገንዘብ ሳይኖሮት ወይም ሳይኖራችሁ ምግብ ያለቀበት ጊዜ ነበረ ወይ?	0	1	
42. ባለፉት ሦስት ወራት ውስጥ በምግብ ወይንም በገንዘብ እጥረት ምክናያት የሚበሉትን የምግቦች አይነት ቁጥር ቀንሰው ያውቃሉ ወይ?	0	1	
43. ባለፉት ሦስት ወራት ውስጥ ልጆችዎ በምግብ ወይንም በገንዘብ እጥረት ምክንያት በቂ ምግብ ያልበሉበት ጊዜ ነበረ ወይ?	0	1	
44. ባለፉት ሦስት ወራት ውስጥ በገንዘብ ወይም በምግብ እጥረት ምክንያት እርሶ ወይንም ቤትዎ ውስጥ ያሉ በቀን የሚበሉት ጊዜዎች ቀንሰው ያውቃሉ ወይ?	0	1	
45. ባለፉት ሦስት ወራት ውስጥ በገንዘብ ወይም በምግብ እጥረት ምክንያት ሙሉ ቀን ያልበሉበትጊዜ ነበረ ወይ? አለ	0	1	
46. ባለፉት ሦስት ወራት ውስጥ ምግብ ለመግዛት ገንዘብ ወይንም ምግብ ጠይቀው ያውቀሉ ወይ?	0	1	
47. የግልዎ የሆነ ቤት አለዎት፣	0	1	
48. ለእርሻ ወይም አትክልት የሚሆን የግል ይዞታ መሬት አለዎት፣	0	1	
49. በቤትዎ ብስክሌት አለ	0	1	
50. ራዲዩስ አለ	0	1	
51. ቤታችሁ ኤሌክትሪክ አለዉ፣	0	1	
52. በሬ አለዎት፣	0	1	
53. ላም አለዎት፣	0	1	
54. ዶሮ አለዎት፣	0	1	
55. ፍየል ወይም በግ አለዎት፣	0	1	
56. ሞባይ ወይም ሴላ ስልክ አለዎት	0	1	
57. ቴሌቪዠን አለዎት	0	1	
58. አህያ አለዎት	0	1	
59. ፈረስ ወይም በቅሎ አለዎት	0	1	
60. ማንጎ፣ ቡና፣ ፓፓያ የመሳሰሉት አለዎት	0	1	
61. ቴፕ	0	1	

62. የቤታችሁ ወለል ምንድን ነዉ	1. አፈር	2. ጣውላ	3.ልሾ/ሲሚንቶ	
63. የቤታችሁ ጣራ ምንድን ነዉ?	1. ቆርቆሮ	2. ሣር	3. ሲምንቶ	
64. የቤታችሁ ግድግዳ ምንድን ነዉ?	1. ጭቃ	2. ጡብ/ሲሚንቶ	3. እንጨት	

65. የመጠጥ ዉሃ የሚያገኙት ከየት ነዉ?	1. ከባንባ	2. ከተጠበቀ ምንጭ ወይም ጉድጓድ	3. ካልተጠበቀ ምንጭ ወይም ጉድጓድ	4. ከወንዝ	
66. ምነ አ ይነት ስንተ ቤት ለለዎት		1. የግል ሰንተ ቤት	2. የጋራ ስንት ቤት	3. ሜዳ ሳይ ነዉ የሚጠቀሙበት	

ክፍል ሠ፡ አስጨናቂ ገጠመኞችን በተመለከተ፤ ከዚህ በፊት ቃለ መጠይቅ ለተደረጉ ባለፈው 6 ወር ውስጥ<br><br>የሚከተሉት ሁኔታዎች ተከስተው እንደሆነ ይጠይቁ ከዚህ በፊት ቃለ መጠይቅ ላልተደረጉ በሂወት ዘመናቸው የሚከተሉት ሁኔታዎች አጋጥማዋቸው እንደሆነ ይጠይቁ				
	የለም	አዎ	ፍቃደኛ አይደሉም	
67. መጠለያ ማጣት	0	1	99	
68. ምግብ ወይም ውሃ ማጣት	0	1	99	
69. ምንም ዓይነት ህክምና ያልተደረገለት ህመም ነበረብዎ	0	1	99	
70. የግል ንብረትዎ መውደም ወይም መወረስ	0	1	99	
71. በጦርነት ወይም ውጊያ ውስጥ መሳተፍ/በተለይም በቦምብና መድፍ የታገዘ/	0	1	99	
72. በአደገኛ ሁኔታ አካባቢውን እንዲለቁ መገደድ	0	1	99	
73. ተደባድበው ያውቃሉ	0	1	99	
74. በጩቤ መወጋት ወይም በለሳ ስለት /ለምሳሌ ገጀራ/ መቆረጥስ	0	1	99	
75. በእስር ወይም ምርኰ የተነሳ አካላዊና አዕምሮአዊ ስቃይ ደርስቦዎታል	0	1	99	
76. በጦርነት ወይም በፍንዳታ የተነሳ ከባድ የአካል ጉዳትስ ደርቦብዎታል	0	1	99	
77. እስር ቤት ታስረው ያውቃሉ	0	1	99	
78. የግዳጅ ስራ /ልክ እንደ እንስሳ ወይም እንደ ባሪያ/ እንዲሰሩ ተደርገው ያውቃሉ	0	1	99	
79. በግዳጅ ወይም በስርቆት ገንዘብዎ ወይም ንብረትዎ ተዘርፎ ያውቃል	0	1	99	
80. አንድን ፅንሰ-ሃሳብ እንዲቀበሉት በሌሎች ግለሰቦች በተደጋጋሚ ተሰብከው ያውቃሉ	0	1	99	
81. ስመሸሽ ሲሉ ተደብቀው ያውቃሉ	0	1	99	
82. መታገት ወይም ታፍኖ መወሰድስ	0	1	99	
83. ከቤተሰብዎ አባላት ተገዶ የመለየት አጋጣሚስ ነበረብዎ	0	1	99	
84. ሬሳን ፈልገው እንዲቀብሩ ተገደው ያውቃሉ	0	1	99	
85. ከሌሎች ሰዎች እንዲለዩ ተገደው ያውቃሉ	0	1	99	
86. ሌላ ሰው እንዲክድዎትና ለሞት ወይም የማቁሰል አደጋ እንዲያጋልጥዎት ተገዶ ያውቃል	0	1	99	
87. የሞተበዎትን ሰው እንዳይቀብሩ ተከልክለው ያውቃሉ	0	1	99	
88. የሙታንን ቅሬት ወይም መካነ-መቃብር እንዲያወድሙ ተገደው ያውቃሉ	0	1	99	
89. በቤተሰብዎ አባል ወይም ጓደኛዎ ሳይ አካላዊ ጉዳት እንዲያደርሱ ተገደው ያውቃሉ	0	1	99	

	የለም	አዎ	ፍቃደኛ አይደሉም	
90. ዘመድ ወይም ጓደኛ ባልሆነ ሰወስ ሳይ አካላዊ ጉዳት እንዲያደርሱ ተገደው ያውቃሉ	0	1	99	
91. የሌላ ሰውን ንብረት ወይም ይዞታ እንዲያወድሙ ወይም እንዲያጠፉ ተገደው ያውቃሉ	0	1	99	
92. የቤተሰብዎ አባል ወይም ጓደኛዎን እንዲከዱ ብሎም ለሞት ወይም ለአካላዊ ጉዳት እንዲያጋልጡት ተገደው ያውቃሉ	0	1	99	
93. በሌላ ሰውስ ሳይ (የቤተሰብዎ አባል ወይም ጓደኛዎ ያልሆነ ሰው) እንዲከዱ ብሎም ለሞት ወይም ለአካላዊ ጉዳት እንዲያጋልጡት ተገደው ያውቃሉ	0	1	99	
94. በግጭት ወይም በረብሻ የትዳር አጋርዎ ተገድሎቦታል	0	1	99	
95. በግጭት ወይም በረብሻ ልጅዎን ተገድሎቦታል	0	1	99	
96. በግጭት ወይም በረብሻ ሌላ የቤተሰብ አባል (ልጅ ወይም የትዳር ጓደኛ ያልሆነ) ተገድሎቦታል	0	1	99	
97. የትዳር ጓደኛዎ ታግተውብዎት ወይም የደረሱበት ጠፍቶ ያውቃል	0	1	99	
98. ልጅዎት ታግተውብዎት ወይም የደረሱበት ጠፍቶ ያውቃል	0	1	99	
99. ሌላ የቤተሰብ አባል (ልጅ ወይም የትዳር ጓደኛ ያልሆነ) ታግተውብዎት ወይም የደረሱበት ጠፍቶ ያውቃል	0	1	99	
100. በጦርነት ወይም በፍንዳታ የተነሳ የቤተሰብዎ አባል ወይም ጓደኛዎ ሳይ የደረሰ ከባድ የመቁሰል አደጋ ነበር ወይ	0	1	99	
101. አንድ ግለሰብ ጭንቅላቱን ወይም ሌላ አካሉን ሲደበደብ አይተው ያውቃሉ	0	1	99	
102. ሰው በግርፋት፡ኤሌክትሪክ ወዘተ በመሳሰሉት ስቃይ ሲደርስበት አይተው ያውቃሉ	0	1	99	

103. ከላይ ከተዘረዘሩት አሰቃቂ ገጠመኞች በተጨማሪ በህይወት ዘመንዎ አጋጥሞዎት የሚያውቅ በጣም አስፈሪ ወይም ለህይወትዎ አስጊ ነገሮች ነበሩ ወይ

ካሉ --

STRESSFUL EVENTS

አስጨናቂ ገጠመኞችን በተመለከተ፤ ቃለ መጠይቅ ለሚደረጉ ሁሉ

የሚከተሉት ሁኔታዎች ባለፈው 6 ወር ውስጥ ተከስተው እንደሆነ ይጠይቁ

	የለም	አዎ	ፈቃደኛ አይደሉም	
104. እርሶ ወይንም ከቤተሰብዎ መሀል ሥራ ማጣት	0	1	99	
105. እርሶ ወይንም አቤት ውስጥ የምኖሩ ቤተሰቦ በጠና መታመም	0	1	99	
106.በገንዘብ እጥረት ምክንያት የለት ተለት አስፈላጊ ነገሮች አለመሳካት	0	1	99	
107. እርሶ ወይንም አቤት ውስጥ የምኖሩ ቤተሰቦ የገንዘብ እዳ ወስጥ መግባት	0	1	99	
108. ከሰው ጋር ተሰዳድቦ መጣላት	0	1	99	
109. እረሶ ወይንም ቤቶ መዘረፍ	0	1	99	
110. ድብድብ ውስጥ መግባት ወይንም ሰው ሲደባደብ መመልከት	0	1	99	

	የለም	አዎ	ፈቃደኛ አይደሉም	
111. ቤተሰቦ ውስጥ ሞት መከሠት	0	1	99	
112. ህግ ከመጣስ የተያያዙ ችግሮች	0	1	99	
113. አዲስ ቤት መግባት	0	1	99	
114. ሌሎች ያጋጠሞት አስጨናቂ ገጠመኞች	0	1	99	

Specifiy ማብራሪያ---

ክፍል ፈ፡ የድህረ-አሰቃቂ ገጠመኝ ምልክቶች

ሰዎች መጥፎ ነገሮች በህይወታቸው ከገጠሟቸው ወይም አሰቃቂ ነገሮችን ከተመለከቱ የሚከተሉት የስነ-ልቦና ችግር ምልክቶች ሲታዩባቸው ይችላሉ፡፡ እያንዳንዱን ምልክት በጥንቃቄ ካደመጡ በኃላ እነዝህ ምልክቶች ባለፈው ሳምንት እርሶን ምን ያህል እንዳስጨነቁዎት ይጠቁሙ፡፡

	በፍፁም	በመጠኑ	በከፍተኛ	በጣም ከፍተኛ	ፍቃደኛ አይደሉም	
115. ባለፈው ሳምንት የአስፈሪ ነገሮች/ድርጊቶች ትውስታ መደጋገም እርሶን ምን ያህል አስጨንቆት ነበር	1	2	3	4	99	
116. ባለፈው ሳምንት አሰቃቂወ ድርጊት ይደገማል ብሎ ማሰብ እርሶን ምን ያህል አስጨንቆት ነበር	1	2	3	4	99	
117. ባለፈው ሳምንት ተደጋጋሚ የሆነ ቅዥት መኖር እርሶን ምን ያህል አስጨንቆት ነበር	1	2	3	4	99	
118. ባለፈው ሳምንት ከሰዎች የመነጠል ስሜት መኖር እርሶን ምን ያህል አስጨንቆት ነበር	1	2	3	4	99	
119. ባለፈው ሳምንት ስሜት ማጣት እርሶን ምን ያህል አስጨንቆት ነበር	1	2	3	4	99	
120. ባለፈው ሳምንት ሆደ-ባሻ መሆን፡ በቀላሉ መከፋት ወይም መቆጣት እርሶን ምን ያህል አስጨንቆት ነበር	1	2	3	4	99	
121. ባለፈው ሳምንት ለነገሮች ትኩረት የመስጠት ችግር መኖር እርሶን ምን ያህል አስጨንቆት ነበር	1	2	3	4	99	
122. ባለፈው ሳምንት እንቅልፍ ማጣት ችግር መኖር እርሶን ምን ያህል አስጨንቆት ነበር	1	2	3	4	99	
123. ባለፈው ሳምንት ሰዎች ይከታተሉኛል ብሎ መስጋት እርሶን ምን ያህል አስጨንቆት ነበር	1	2	3	4	99	
124 ባለፈው ሳምንት. ግልፍተኛ ወይም ነጭናጫ በመሆኖ ምን ያህል አስጨንቆት ነበር	1	2	3	4	99	
125. ባለፈው ሳምንት አሰቃቂ ድርጊቶችን ሊያስታውሱ የሚችሉ ነገሮችን /ድርጊቶችን/ ለመከላከል ይሞክሩ ነበር	1	2	3	4	99	

	በፍፁም	በመጠኑ	በከፍተኛ	በጣም ከፍተኛ	ፍቃደኛ አይደሉም	
126. ባለፈው ሳምንት አሰቃቂ ድርጊቶችን መይም ሁኔታዎች ለማስታወስ አለመቻል ምን ያህል አስጨንቆት ነበር	1	2	3	4	99	
127. ባለፈው ሳምንት በዕለታዊ ክንዋኔዎች ላይ ብዙም ፍላጐት አለማሳየ እርሶን ምን ያህል አስጨንቆት ነበር	1	2	3	4	99	
128. ባለፈው ሳምንት የወደፊት ተስፋ ማጣት ስሜት እርሶን ምን ያህል አስጨንቆት ነበር	1	2	3	4	99	
129. ባለፈው ሳምንት መጥፎ ገጠመኝን ሲያስታውሱ የሚችሉ ሃሣቦች ወይም ስሜቶችን ለማስወገድ ሞክረው ነበር	1	2	3	4	99	
130. ባለፈው ሳምንት መጥፎው/ዘግናኙ ድርጊት ሲታወስዎ የሚያሳዩት መንፈሳዊና አካላዊ ለውጥ መኖር እርሶን ምን ያህል አስጨንቆት ነበር	1	2	3	4	99	
	በፍፁም	በመጠኑ	በከፍተኛ	በጣም ከፍተኛ	ፍቃደኛ አይደሉም	
131. ባለፈው ሳምንት ቀድሞ ከነበረዎ ችሎታ የቀነሰ መምሰል እርሶን ምን ያህል አስጨንቆት ነበር	1	2	3	4	99	
132. ባለፈው ሳምንት ከአዳዲስ ሁኔታዎች ጋር ራስን በቀላሉ የማላመድ ችግር እርሶን ምን ያህል አስጨንቆት ነበር	1	2	3	4	99	
133. ባለፈው ሳምንት የድካም ስሜት መኖር እርሶን ምን ያህል አስጨንቆት ነበር	1	2	3	4	99	
134. ባለፈው ሳምንት የሰወነት ሕመም ስሜት መኖር እርሶን ምን ያህል አስጨንቆት ነበር	1	2	3	4	99	
135. ባለፈው ሳምንት አካላዊ መታወክ እርሶን ምን ያህል አስጨንቆት ነበር	1	2	3	4	99	
136. ባለፈው ሳምንት የማስታወስ ችሎታ መቀነስ እርሶን ምን ያህል አስጨንቆት ነበር	1	2	3	4	99	
137. ባለፈው ሳምንት ቀደም ብሎ የሠሩትን ሥራ ሌላ ሰው እስኪነግረዎት ድረስ አለማስታወስ ምን ያህል አስቸግሮት ነበር	1	2	3	4	99	
138. ባለፈው ሳምንት በነገሮች ላይ አትኩሮት ያለመስጠት ችግር እርሶን ምን ያህል አስጨንቆት ነበር	1	2	3	4	99	
139. ባለፈው ሳምንት ሁለት ስብዕና ኖርዎት፡ አንዱ እርሶዎ የሚያደርገትን ይመለከታል/ ይከታተላል/ እርሶን ምን ያህል አስጨንቆት ነበር	1	2	3	4	99	
140. ባለፈው ሳምንት የዕለታዊ ክንዋኔዎችን ማቀድ አለመቻል እርሶን ምን ያህል አስጨንቆት ነበር	1	2	3	4	99	
141. ባለፈው ሳምንት ለሆነው ነገር ሁሉ ራስዎን ይወቅሱ ነበር	1	2	3	4	99	
142. ባለፈው ሳምንት ከመጥፎው አደጋ በመትረፍዎ የሚሰማዎ የፀፀት ስሜት እርሶን ምን ያህል አስጨንቆት ነበር	1	2	3	4	99	
143. ባለፈው ሳምንት ተስፋ መቁረጥ ስሜት እርሶን ምን ያህል አስጨንቆት ነበር	1	2	3	4	99	

	በፍፁም	በመጠኑ	በከፍተኛ	በጣም ከፍተኛ	ፍቃደኛ አይደሉም	
144. ባለፈው ሳምንት በገጠመዎ አሰቃቂ ድርጊት የተነሳ የመሸማቀቅ/ማፈር ስሜት እርሶን ምን ያህል አስጨንቆት ነበር	1	2	3	4	99	
145. ባለፈው ሳምንት የደረሰብዎትን ችግር ሰዎች አይረዱልኝም ብለው ማሰብ እርሶን ምን ያህል አስጨንቆት ነበር	1	2	3	4	99	
146. ሰዎች ይጠሉኛልን የሚል ስሜት መኖር እርሶን ምን ያህል አስጨንቆት ነበር	1	2	3	4	99	
147. ባለፈው ሳምንት የሚተማመኑበት ወይም ሃሣብዎን የሚያካፍሉት ሰው ያለዎ አይመስሎትም ነበር	1	2	3	4	99	
148. የሚያምኗቸው ሰዎች የከዱዎት ይመስልዎታል	1	2	3	4	99	
149. ባለፈው ሳምንት ባጋጠመዎ ነገር የተነሳ የበታችነት ስሜት መኖር እርሶን ምን ያህል አስጨንቆት ነበር	1	2	3	4	99	
150. በሌሎች ሰዎች ያልታመኑ ምን ያህል ይመስሎት ነበር	1	2	3	4	99	
151. ሌሎችን ለማገዝ ብርታት የማጣት ስሜት	1	2	3	4	99	
152. ባለፈው ሳምንት መጥፎ ድርጊት/ቶች ለምን ሲደርሱብዎ እንደቻሉ ብዙ ጊዜ ማሰላሰል	1	2	3	4	99	
153. በዘግናኝ ድርጊቶች ውስጥ ያለፉ እርሶ ብቻ እንደሆኑ ማመን/ማሰብ	1	2	3	4	99	
154. ባለፈው ሳምንት የበቀለኝነት ስሜት መኖር እርሶን ምን ያህል ተፈታትኖት ነበር ፣	1	2	3	4	99	

ክፍል ሸ፡ የአዋቂ ሰውን የነርቭ ችግርን በተመለከተ

	አይደለም	አዎ	አላውቅም	ፍቃደኛ አይደለሁም	
155. በአደጋ የተነሳ ወይም ጭንቅላትዎን ተመተው ራስዎን ስተው ያውቃሉ	0	1	88	99	
156. እጅዎን የመደንዘዝ ስሜት አለብዎ	0	1	88	99	
157. እግርዎን የመደንዘዝ ስሜት አለብዎ?	0	1	88	99	
158. የሚጥል በሽታ ኖሮት ያውቃሌ	0	1	88	99	
159. አልፎ አልፎ የሚከሰት የክንድ መዛል/መድከም/ አጋጥሞት ያውቃል	0	1	88	99	
160. አልፎ አልፎ የሚከሰት እግርዎ መዛል/መድከም/ አጋጥሞት ያውቃል	0	1	88	99	
161. በክንድዎ ወይም በእጅዎ ላይ ቋሚ የሆነ ድካም ወይም ዝለት አለብዎ	0	1	88	99	
162. በፊተዎ ላይ ቋሚ የሆነ ዝለት/መዛባት አለብዎ	0	1	88	99	
163. በጫማዎ ወይም በእግርዎ ላይ የቆዩ ዝለት/ድካም አለብዎ	0	1	88	99	
164. የዓይን ብዥታ አለብዎ	0	1	88	99	

165. ስአፍታ የሚቆይ የዓይን ግርዶሽ ኑሮት ያውቃል	0	1	88	99	
166. እግርዎ ወይም እጅዎ አንዳንዴ ተንቀጥቅጦ ያውቃሌ	0	1	88	99	
167. ዕቃዎን ለማንሳት ወይም ለመያዝ ሲሞክሩ እጅዎ እየተንቀጠቀጠ አስቸግሮዎት ያውቃሌ	0	1	88	99	
168. የመናገር ችግር አጋጥሞት ከጊዜ በኋላ ደህና የሆኑበት አጋጣም ነበር	0	1	88	99	
	አይደለም	አዎ	አላውቅም	ፍቃደኛ አይደለሁም	
169. ለረጅም ጊዜ የቆየ የመናገር ችግር አለብዎ	0	1	88	99	
170. ሲሄዱ ያነክሳሉ	0	1	88	99	
171. ሁል ጊዜ የሚኖር የወገብ ህመም አለብዎ	0	1	88	99	
172. ሁል ጊዜ የሚኖር አንገት ህመም አለብዎ	0	1	88	99	
173. ለደ ፊትዎ ላይ የሚሰራጭ ህመም አለብዎ	0	1	88	99	
174. ሲቆጣጠሩት የማይችሉት መንቀጥቀጥ/መውረር/ በፊትዎ ላይ አለብዎ	0	1	88	99	
175. ከእንቅልፍዎ የሚቀሰቅስ የእጅ ህመም ኖሮት ያውቃሌ	0	1	88	99	
176. ተደጋጋሚ የሆነ የሽንት ወይም ሠገራ መቆጣጠር ችግር አለብዎ	0	1	88	99	

177. እዚ የሚታየው የሥዕላዊ መግለጫ መሰላል በእርሶ ቀበሌ ውስጥ ነዋሪዎችን እንደሚመስል ያስቡና.
እሱን በተመለከተ ጥያቄ እጠይቆጣለው::

ሥዕሉ መሰላል ጫፍ የሚያመለክተው በዚ ቀበሌ ውስጥ ያሉ ሀብትም -ገንዘብና ቤት ያላቸውን ያመለክታል. መሰላሉ ሥር ደሞ በዚ ቀበሌ ውስጥ ያሉ ድሃ ወይንም ገንዘብና ቤት የሌላቸውን ሰዎችን ይመለከታል:: ወደ መሰላሉ ጫፍ መቅረብ ወደ ገንዘብ ወይንም ቤት ያላቸው ሰዎችን መቅረብ ያመለክታል:: ወደ መሰላሉ ሥር መቅረብ ወደ ደሞ ድሃነት ወይንም ገንዘብና ቤት የሌላቸውን ሰዎችን መቅረብን ያመለክታል::

ባሁኑ ጊዜ የሕወቶ ደረጃ በቀበሌዎት ካሉ ሰዎች ጋር ሲነጻጸር ብለው የሚያስቡትን የሥዕሉ መሰላል ደረጃ ላይ በ X ያስምሩበት :: ቁጥሩን በወረቀቱ ያስፍሩበት

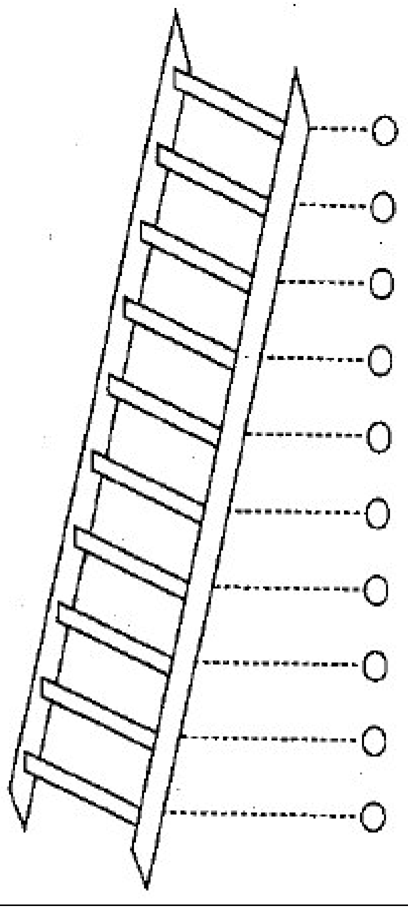

178. የተለያዩ የምርጫ ሙከራዎችን በተመለከተ:: **DISCRETE CHOICE EXPERIMENT**

አሁን ስምንት ካርዶች አሳዮታለሁ:: እያንዳንዱ ካርድ ስለ ሁለት ጤና ጣቢያ የሚገልጽ ነው:: አሁን በጠና ቢታመሙ ብለው ያስቡና የትኛው የጤና ጣብያ ውስጥ ቢታከሙ እንደሚመርጡ ይንገሩኝ:: ሁለቱንም ጤና ጣብያ አለመጠቀም ምርጫ አሎት:: በነዚህ ጥያቄዎች ሳይ ትክክል ወይንም የተሳሳተ መልስ የለም:: ከነዘሂ ጥያቄዎች ማወቅ የምንፈልገው ምን ያህል ጤና ጣብያ ውስጥ መታከም አስፈሳጊ ነው ብለው የሚያምኑበትን ነገሮች ለማዮት ነው::

ሁለቱንም ጤና ጣብያ አለመጠቀም ምርጫ አሎት:: በዚህ ጥያቄ ሳይ ትክክል ወይንም የተሳሳተ መልስ የለም::

	ጤና ጣብያ (ሀ)	ጤና ጣብያ (ለ)	ወደ ሁሉቱመ አለሄድም	ፍቃደኛ አይደለሁም
ካርድ1	1	2	3	99
ካርድ2	1	2	3	99
ካርድ3	1	2	3	99
ካርድ4	1	2	3	99
ካርድ5	1	2	3	99
ካርድ6	1	2	3	99
ካርድ7	1	2	3	99
ካርድ8	1	2	3	99

ከነዚ ጥያቄዎች ማወቅ የምንፈልገው ምን ያህል ጤና ጣብያ ውስጥ አስፈሳጊ ብለው የሚያምኑበትን ነገሮች ለማይት ነው::

ለኢንተርቭው ለምያደርገው ሰው:: የተጠቀሙትን የካርድ አይነቱን ቁጥር ያስምሩበት 1 2 3 4 5
INTERVIEWER: PLEASE CIRCLE THE VERSION NUMBER USED: 1. 2. 3. 4. 5

መጠነሰብ				
አባት	ክብደት		ቁመት	

አመሰግናለሁ!!
